# Evidence supporting the first secondary chromosome in Actinobacteria as a hallmark of the Embleya genus

**DOI:** 10.1099/mgen.0.001704

**Published:** 2026-05-07

**Authors:** Juan Pablo Gomez-Escribano, Siobhán Dorai-Raj, David Baker, Ernest Lacey, Barrie Wilkinson, Thomas J. Booth

**Affiliations:** 1 Molecular Microbiology, John Innes Centre, Norwich Research Park, Norwich NR4 7UH, UK; 2 Leibniz-Institut DSMZ – German Collection of Microorganisms and Cell Cultures, Inhoffenstraße 7B, 38124 Braunschweig, Germany; 3 Centre for Microbial Interactions, Norwich Research Park, Norwich NR4 7UG, UK; 4 Earlham Institute, Norwich Research Park, Norwich NR4 7UZ, UK; 5 Microbial Screening Technologies Pty. Ltd., Smithfield, NSW 2164, Australia; 6 The Novo Nordisk Foundation Center for Biosustainability, Danmarks Tekniske Universitet, Kongens Lyngby 2800, Denmark

**Keywords:** chromid, *Embleya*, evolution, megaplasmid, secondary chromosome, *Streptomycetaceae*

## Abstract

*Embleya* is a genus within the family *Streptomycetaceae*, a group of Actinobacteria with an outstanding capacity for the production of specialized metabolites, and a strikingly complex life cycle. In this work, we sequenced the complete genome of the new species *Embleya australiensis* MST-111070 and validated the assembly using optical mapping. The genome of *E. australiensis* MST-111070 consists of a 7.1 Mb linear chromosome and three additional replicons (EEC, *Embleya* extrachromosomal element), including a 4.2 Mb linear replicon, EEC1, significantly larger than all previously described secondary replicons from bacteria. EEC1 is typified by its similar composition to the chromosome in terms of G+C content, codon usage and gene functions. It also carries terminal inverted repeats identical to the chromosome. EEC1 is enriched in biosynthetic gene clusters (BGCs), including the only copy of the BGCs for the spore pigment and the surfactant peptide SapB, metabolites essential for the organism’s life cycle. EEC1 contains an origin of replication with at least some chromosomal properties, and its replication is likely to depend on functions provided by chromosomally located genes. Further comparison of *Embleya* spp. genomes suggests that EEC1-like replicons are conserved across the genus, in contrast to other known large linear extrachromosomal replicons (megaplasmids) in the family *Streptomycetaceae*. EEC1 is thus a hallmark of the *Embleya* genus and is central to its evolution within the *Streptomycetaceae* family. We propose EEC1 as a secondary chromosome and the largest secondary replicon reported in bacteria to date.

**Impact Statement**

Bacteria encode multipartite genomes, typically consisting of a single chromosome and auxiliary plasmids. Some *Pseudomonadota* (Proteobacteria), *Cyanobacteriota* (Cyanobacteria) and *Deinococcota* (Deinococcii) species additionally encode large secondary chromosomes. In this study, we propose the first secondary chromosome from *Actinomycetota* (Actinobacteria), EEC1 from *Embleya australiensis* MST-111070, after confirmation of the replicon through optical mapping and genome sequencing. We show that EEC1-like replicons are conserved across *Embleya* species and represent a defining feature of the genus. At 4.2 Mb, EEC1 represents the largest confirmed secondary replicon in bacteria. Given their size and the genus-specificity of encoded genes, the emergence of EEC1-like replicons represents a significant event in the evolution of the *Streptomycetaceae*. EEC1-like replicons upend our understanding of multipartite genomes and provide a valuable reference point for future comparative studies.

## Data Summary

The data for the *Embleya australiensis* MST-111070 genome assembly are available on NCBI as BioProject PRJNA1219506 and BioSample SAMN46558982; the genome assembly can be accessed under GenBank Accessions CP182422–CP182425. The Illumina and PacBio reads have been deposited in NCBI SRA under accession codes SRX29683700–SRX29683701 and SRX29688493–SRX29688495, respectively. Data that could not be deposited in the NCBI databases are freely available on Zenodo: Bionano original raw data and BNX molecules, together with the same genome assembly in GenBank format, are available at https://zenodo.org/records/15915371, and PacBio assembled reads in original .h5 format are available at https://zenodo.org/records/15961674. The following software was developed as part of this project and is publicly available (in these repositories) under GPL-3.0 licence: codoniser (https://github.com/drboothtj/codoniser), egger (https://github.com/drboothtj/egger) and gcskewer (https://github.com/drboothtj/gcskewer).

## Introduction

Actinobacteria (*Actinomycetota*) are a diverse and important phylum of bacteria, including major pathogens, rhizobionts and producers of bioactive natural products, including essential medicines. Actinobacteria play host to a wide variety of extrachromosomal elements, namely plasmids [1,2] and megaplasmids [3,5]. These extrachromosomal replicons play an important role in virulence [6,7], resistance [8] and biosynthesis [3]. Exploiting rare and under-represented Actinobacteria is an important strategy for drug discovery [9]. Moreover, exploring these genomes will also lead to breakthroughs in fundamental biology of the phylum.

Within the phylum *Actinomycetota* is the family *Streptomycetaceae*. Members of this family possess one of the most complex life cycles among bacteria [10,11]. Briefly, monogenomic spores germinate into multigenomic (coenocytic) vegetative mycelium that, in turn, differentiates into aerial hyphae. These hyphae undergo compartmentalization into the spores as means of reproduction, dispersion and resistance against environmental conditions. This complexity is mirrored in the genome. *Streptomycetaceae* encode large, linear chromosomes (typically 7–12 Mb) [12] that are often complemented with auxiliary plasmids and megaplasmids, ranging in size from around 6 Kb to 1.8 Mb [13,14]. The larger of these replicons are reminiscent of the secondary chromosomes of *Proteobacteria* [15,16], given their large relative size and their ability to encode ‘core’ genes. However, these large replicons fall short of this classification, as they are not conserved outside the species and are apparently non-essential [13]. This demonstrates the unique nature of secondary replicons from the *Streptomycetaceae* (a summary of the definitions used in this study is shown in Table 1).

**Table 1.** Distinguishing features of chromosomes, secondary chromosomes, chromids and megaplasmids Adapted from Harrison *et al.* [15] and DiCenzo *et al.* [2], and does not include the observations and discussion from this study. Relative terms are given as objective measures vary highly between bacterial phyla. Depending on literature, chromid and secondary chromosome are used interchangeably.

Feature	Megaplasmid	Chromid (secondary chromosome)	Secondary chromosome (non-chromid, this study)	Chromosome
Observed in bacteria	Yes	Yes	Yes	Yes
Core genes	No	Yes	Yes	Yes
Nucleotide composition	Variable	As chromosome	As chromosome	Variable
Codon usage bias	Average	High	Highest	Highest
Replication system	Plasmid	Plasmid	Incomplete-chromosomal	Chromosomal
Gene origins	Strain-specific	Genus-specific	Genus-specific	Less genus-specific
Evolutionary origin	Plasmid	Plasmid	Unknown	Chromosome

*Embleya* is a genus of the *Streptomycetaceae* family, proposed after the reclassification of *Streptomyces scabrisporus* to *Embleya scabrispora* [17,18], the type species of the genus, in 2018 [17]. At the time of writing, the genus contains only one other named species, *Embleya hyalina* (as stated in the LPSN database [19], last accessed on 8 October 2024). *E. scabrispora* and *E. hyalina* are known to produce the specialized metabolites hitachimycin [20,21] and nybomycin [22,23], respectively. Several additional strains have been identified by 16S sequencing, but taxonomic descriptions are poor, with *Embleya* strains being erroneously described as *E. scabrispora* [24]. The NCBI’s refseq database contains few *Embleya* genomes, most of which are relatively low-quality assemblies (N50<1 Mb). During this study, two additional high-quality sequences were published alongside over 1,000 additional actinobacterial genomes [12], but the characterization of these strains was limited. A recent bioinformatic analysis has revealed the diversity and breadth of biosynthetic gene clusters (BGCs) present in *Embleya* genomes [24]. However, the lack of complete *Embleya* genomes severely restricts the analysis. Given the potential value of *Embleya* spp., there is significant need for high-quality *Embleya* genomes to provide a foundation for exploring this enigmatic genus.

Here, we describe the genome of a new species, *Embleya australiensis* MST-111070, a known producer of nybomycins, leptomycins, kazusamycins and antibiotic L-156602. We describe the presence of a giant 4.2 Mb secondary replicon and confirm its existence through optical mapping. Finally, we conduct an in-depth bioinformatic analysis of the secondary replicon and provide evidence for an essential role during the evolution of the genus, leading to the proposal for the classification of this replicon as a secondary chromosome.

## Methods

### Strains and culture conditions

*E. australiensis* MST-111070 was isolated from arid soil collected in South Australia in 1996. Cultivation was performed on SFM agar (20 g l^−1^ Soya Flour, 20 g l^−1^ Mannitol) [10] for sporulation; spore stocks were prepared following established methods [10].

### DNA extraction and quality control

High molecular weight DNA samples were extracted from early-stationary-phase liquid cultures in 1:1 TSB:YEME medium [10], following the salting-out protocol [10]. Sample quality was first assessed by standard agarose-gel electrophoresis and pulse-field gel electrophoresis. Quantification and further quality control were performed with NanoDrop and Qubit (Thermo Fisher Scientific) following manufacturers’ instructions, before handing the samples to sequencing providers.

### Genome sequencing and assembly

PacBio single-molecule, real-time (SMRT) sequencing and assembly (Pacific Biosciences of California, Inc.) was commissioned to the Earlham Institute (Norwich Research Park, Norwich, NR4 7UZ, UK). Sequencing was performed with C4-P6 chemistry on three SMRT cells with an RSII sequencer; the data were processed and assembled with HGAP.2 and HGAP.3. Illumina sequencing and assembly was commissioned to the DNA sequencing facility in the Department of Biochemistry, University of Cambridge (Cambridge, CB2 1GA, UK). Details given by the sequencing provider: a shotgun library with an average insert size of 550 bp was prepared using the Illumina TruSeq PCR-free/Nano library preparation kit; a second shotgun library with an average insert size of 4 kb was prepared using the Illumina Nextera Mate Pair library preparation kit; both libraries were sequenced using V2 Illumina sequencing chemistry and run on a MiSeq instrument (2×250 bp paired-end sequencing). Sequence data were processed and assembled with a custom pipeline developed at the facility (Dr Markiyan Samborskyy, unpublished data) and as previously reported [25].

### Optical mapping

Optical mapping was performed with Bionano Irys (Bionano Genomics, San Diego, USA) technology. Bacterial cells were prepared from a liquid culture following supplier’s instructions; DNA extraction, labelling and data collection, including processing of images to extract BNX files with molecules information, were commissioned to the Earlham Institute (Norwich Research Park, Norwich, NR4 7UZ, UK). We obtained BNX files with Bionano molecules information for a high-resolution map of the distribution of the recognition site for nickase Nt.BspQI (GCTCTTC). The BNX files were processed with Bionano’s software IrysView Genomic Analysis Viewer, version 2.5.1.29842, with versions r5134 of PipelineCL.py, r5122 of RefAligner.cpp, r5122 of Assembler.cpp and r5146 of Hybrid Scaffold; all running on Microsoft Windows 1,064 bits with Python 2.7.8. The software was used following the guidelines in the Bionano document ‘IrysView^®^ v2.5.1 Software Training Guide. Document Number: 30035 Document Revision: G, 2016’. The result of Molecule Quality Report (alignment over reference sequence) was interpreted according to the document ‘Guidelines for Interpreting the Bionano Molecule Quality Report. Document number 30175, Rev A’. Our analysis strongly benefited from invaluable advice provided by BioNano Technical Support. A detailed account of the methodology is given in Supporting Information.

### Sequence data analysis

General visualization, analysis and manipulation of DNA sequence data were performed with computer programs Artemis [26], Artemis Comparison Tool [27] and NotePad++ (http://notepad-plus-plus.org/). Alignments and assembly of sequences were performed with Staden Package [28,29]. Similarity searches were performed with blast+ [30] at the NCBI web server (http://www.ncbi.nlm.nih.gov/blast/) or on a standalone computer with prfectblast 2.0 [31]. Annotation of gene function and genetic features was performed with antiSMASH (version 7 with ‘relaxed strictness’ and all options enabled) [32] and RAST (Rapid Annotation using Subsystem Technology) [33] using Prodigal [34] for CDS calling and with frameshift fixing and metabolic model building options. Mauve [35] was used for full genome comparisons and contigs reordering. Benchmarking universal single-copy orthologs (BUSCO) [36] was used to analyse the presence of orthologous genes per taxonomic group (run last time on 19 March 2023, version BUSCO v5.4.4). Dot plots were generated using dotplotter v1.0.0 [37]. Orthologous protein detection was performed with the European Galaxy server [38], using the module Proteinortho with default parameters [similarity comparison algorithm: Diamond sensitive (amino acid sequences); Minimal reciprocal similarity in %: 95; Minimal algebraic connectivity: 0.1; E-value threshold of the blast algorithm: 0.001; Minimal coverage of best blast alignments in %: 50; Minimal percent identity of best blast hits in %: 25; Apply self-blast, detects paralogs without orthologs (not compatible with synteny): false; Report singleton genes without any hit: false; Stop clustering if a split would result in groups that do not span across all species of the initial connected component: false; Use isoform information: Don't use isoform information; Activate synteny feature (POFF): false].

### Taxonomic analysis

Type strain genome server (TYGS) [39] and genome-to-genome distance calculator (GGDC) [40] were used for genome-based and 16S rDNA-based taxonomical studies (with extended maximum likelihood and maximum parsimony 16S rRNA analysis, which includes type strains for which the genome sequence is not available). All taxonomical investigations, as well as genome alignments with Mauve [35], were last repeated during May and June 2023 prior to the finalization of this manuscript, as to reflect the most current state of genome and 16S rDNA sequence availability in the databases. The 11-locus tree was produced by running getphylo [41] on 515 complete genomes accessed from the NCBI (21 February 2024) and visualized using iTOL [42].

### Functional and compositional analysis

G+C content and GC skew were calculated with a custom Python package (gcskewer) developed for this project (https://github.com/drboothtj/gcskewer). Codon usage and codon usage correlation were calculated using a Python package (codoniser) developed for this project. The codoniser package is open-source and is available on the Python package index (PyPI) and GitHub (https://github.com/drboothtj/codoniser). Replicon function was estimated using clusters of orthologous gene (COG) categories as annotated in the EggNOG 5.0 [43] database and implemented in eggnog-mapper v2 [44] with the taxonomic scope set to ‘Actinobacteria’. COG category correlation was calculated using a Python package (egger) developed for this project and available on PyPI and GitHub (https://github.com/drboothtj/egger). The rank-specificity of genes was calculated using diamond blastp searches against the predicted proteomes of the complete genomes used in the taxonomic analyses above. Specificity scores were calculated for each protein coding sequence using the following equation:


$$\frac{f{\left(A\right)}^{2}}{f\left(A\right)\text{+}\;f\left(B\right)}$$

Where *f*(A) is the frequency of the gene occurring on a genome in group A and *f*(B) is the frequency of the gene occurring on a genome in group B. For strain-specificity, group A contained only MST-111070 and group B included all other taxa. For genus-specificity, group A contained all the *Embleya* sequences and group B included all other taxa. Finally, for family-specificity, group A contained all the *Embleya*, *Streptomyces*, *Kitosatospora* and *Yinghuangia* sequences and group B included all other taxa.

## Results and discussion

### *E. australiensis* MST-111070 contains a 4.2 Mb second replicon

#### The complete genome of *E. australiensis* MST-111070

Strain MST-111070 was isolated by the biotechnology company Microbial Screening Technologies (Smithfield, Australia) as part of a programme to identify talented, free-growing Actinomycetes from arid soils. Agar cultivation of the strain appeared morphologically as a typical streptomycete that, on methanolic extraction and LCMS, showed high-level production of several rare, specialized metabolites: nybomycin and deoxynybomycin; leptomycin A and B and kazusamycin A and B; and antibiotic L-156602 (Microbial Screening Technologies’ BioAustralis metabolite catalogue and unpublished data). Following the genomic sequencing performed as part of this work, and the taxonomic analysis detailed below, we have reclassified the strain as *E. australiensis* sp*.* nov.

High molecular weight DNA was extracted from *E. australiensis* MST-111070 and sequenced with Pacific Biosciences RSII SMRT technology (PacBio) at the Earlham Institute (Norwich, UK). In total, we attained over 900 Mb of raw sequence data, corresponding to ~90× coverage of an average *Streptomyces* genome. The genome was assembled using HGAP2 and HGAP3 independently (Table 2). Both assemblies were essentially equivalent, consisting of three contigs of 7.1, 4.2 and 0.2 Mb. The HGAP3 assembly included an extra contig of 0.3 Mb. It must be noted that both assemblies originate from the same raw data; therefore, any difference is accounted for exclusively by the changes in the algorithms from version 2 to 3 of the assembly pipeline.

**Table 2.** Comparison of the assemblies generated in this study The size of the *E. australiensis* MST-111070 chromosome and each EEC1–3 is shown in bp. Version 4 was submitted to NCBI.

Assembly version	Method	Chromosome	EEC1	EEC2	EEC3	Total
1	Pacific Biosciences RSII, HGAP2	7,100,744	4,187,648	–	228,860	11,517,252
2	Pacific Biosciences RSII, HGAP3	7,101,142	4,187,746	302,592	228,392	11,820,375
3	Illumina MiSeq, University of Cambridge	7,075,274 21,156	4,164,320	–	207,392	11,468,142
4	Pacific Biosciences RSII and Illumina consensus	7,117,762	4,217,944	302,592	210,636 (circular)	11,848,934

The presence of the large 4.2 Mb contig led us to consider that our assembly might not accurately represent the genome. Therefore, we sought to obtain an independent *de novo* assembly using a different sequencing technology. To achieve this, we turned to Illumina MiSeq using paired-end sequencing, which was, crucially, combined with a Nextera Mate Pair shotgun library and the in-house assembly pipeline at the University of Cambridge DNA sequencing facility (Department of Biochemistry) [25]. This approach produced an assembly consisting of a set of contigs that were very similar to that obtained with PacBio HGAP2, including the 4.2 Mb contig (Table 2).

Subsequently, the contigs from both PacBio assemblies were aligned with the Illumina contigs, and the alignment was manually curated. The Illumina contigs also allowed the extension of the ends of the PacBio contigs (for a similar strategy, see [13,45]) to generate a final combined assembly of four contigs: 7.1 and 4.2 Mb of linear topology; 0.3 Mb apparently linear according to optical mapping, see below; and 0.2 Mb of circular topology (Table 2). These contigs representing the chromosome and the extrachromosomal elements (here designated *Embleya*extrachromosomal elements, EEC1 to 3) were submitted to GenBank with accessions CP182422–CP182425.

#### Terminal inverted repeats in the *E. australiensis* MST-111070 genome

**Fig. 1.**
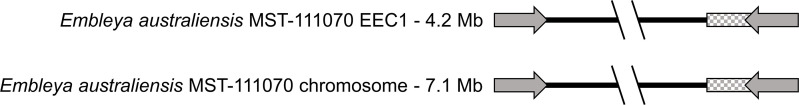
Relationship between the *E. australiensis* MST-111070 chromosome and EEC1. The 21.2 kb TIRs are shown as solid grey arrows, and the 12.4 kb block of additional shared homology is shown as grey checked bar.

In addition to linear chromosomes, many other members of the *Streptomycetaceae* family host additional linear extrachromosomal replicons, all of which contain characteristic terminal inverted repeats (TIRs) [13,46]. TIRs are large regions of DNA (typically 20 Kb–1 Mb) [1,12, 47] found at both ends of the replicon and are required for the replication and stability of linear replicons [46,48,50]. The presence of TIRs in an assembly is usually indicative of a nearly complete replicon sequence. Analysis of the 7.1 Mb contig revealed the presence of TIRs of around 21.2 kb. Interestingly, the TIRs of the 4.2 Mb replicon are almost identical to those of the chromosome (99.95% identity). Furthermore, the homology between the right ends of the genome and EEC1 extends to 33.6 kb of sequence with 99.94% identity (see Figs 1 and S1, available in the online Supplementary Material for further details). This extraordinary share of replicon-end asymmetry could be explained by a hypothetical origin of EEC1 as a schism event from the chromosome, followed by homogenization among the ends of both replicons, a commonly observed mechanism for the maintenance of TIRs [46,49]. The presence of TIRs in EEC1 is strong evidence that it is a standalone replicon, but, given the extraordinary size of EEC1, we sought further independent evidence to confirm its existence and structure as assembled by both DNA sequencing technologies.

#### Confirmation of genome assembly and replicon topology with optical mapping

The genome assembly suggested the existence of a large (4.2 Mb) secondary replicon, which, if confirmed, would be the largest secondary replicon reported from Actinobacteria and of a size comparable to the *Escherichia coli* chromosome. With whole-genome sequencing, there is always the possibility that the sequence is misassembled into artefactual contigs that are not representative of the actual genome structure [51], which could be responsible for the apparent split of the expected chromosome into two contigs. Given the unique nature of the EEC1 replicon, we sought to confirm these results through an independent method.

Optical mapping is a technology that produces high-resolution genome maps of specific sequence motifs recognized by nickase enzymes (see Methods and Supporting Information for a detailed explanation of the technology and methodology). Briefly, high molecular weight fragments of genomic DNA (up to 0.5 Mb) are treated with a nickase that recognizes a specific 6 bp DNA motif and adds a fluorescent tag. An accurate contiguous map of the nickase sites is obtained by high-resolution imaging of individual DNA molecules in a linear ordered fashion. These maps can be used for a *de novo* assembly into contigs representing a consensus linear distribution of the nickase sites along the entire genome, in a similar way to a classical restriction DNA map. Alternatively, the contigs or the individual molecule maps can be aligned to the predicted distribution of the nickase DNA motif along a reference DNA sequence. Because of the large size of the molecules (with sizes exceeding 500 kb in some cases), optical mapping provides a highly accurate and reliable way of checking the accuracy of a DNA sequence assembly and to resolve difficult repeating regions like the TIRs [13,52, 53]. We previously showed the application of this technique to confirm an assembly of the *Streptomyces clavuligerus* ATCC 27064 genome [13].

Here we employed Bionano optical mapping technology to obtain a high-resolution map of the recognition site for nickase Nt.BspQI (GCTCTTC) along the genome. The analysis accounted for 229,509 molecules totalling 37288.4 Mb with a molecule N50 of 158.5 Kb and over 2,000 molecules larger than 500 kb. We used the data to confirm the DNA assembly through two independent methods: (i) an alignment of Bionano molecules to the Illumina-PacBio hybrid assembly; and (ii) generation of a reference-independent *de novo* assembly of Bionano molecules. First, the molecules-to-reference alignment generated statistics indicators that supported an overall reliable alignment of Bionano molecules to the DNA assembly (Map Rate, 42.7%, FP 0.48/100 kbp, FP 4.4% and FN 18.9%; see Methods and Supporting Information for details and documentation on interpretation of results). The molecules aligned with very high confidence, including along the TIRs (Figs S2–S4), providing strong support for the 4.2 Mb contig being a true replicon independent from the 7.1 Mb chromosome. Second, the Bionano *de novo* assembly was performed without the reference genome and the resulting assembly consisted of eight contigs spanning a total of 14,359 kb, with N50 of 4,330 kb and an average coverage of 106.3 molecules. Five contigs mapped to the chromosome, while the remaining three mapped to each of the extrachromosomal elements; in all cases, the DNA contigs were fully covered by the Bionano contigs (see Extended Methods in Supporting Information and Figs S3 and S4). The ends of the DNA contigs were fully supported by the Bionano *de novo* assembled contigs. In addition, the contig matching EEC3 supported the proposed sequence and circular topology (Fig. S4). Furthermore, the Bionano analysis confirmed the existence of EEC2, which was previously not assembled in the Illumina and the HGAP2 assemblies and supports a linear topology of this replicon (Table 1, Fig. S3). Taken together, the DNA sequencing data and optical mapping confirm the genome of *E. australiensis* MST-111070 as follows: a 7.12 Mb linear chromosome; two plasmids: one large circular plasmid (211 kb, EEC3) and another large linear plasmid (303 kb, EEC2); and an extraordinary 4.2 Mb linear replicon (EEC1).

### Taxonomy of *E. australiensis* MST-111070

Phylogenetic analysis of the genome of MST-111070 indicated this strain is potentially a new species within the *Embleya* genus, for which we propose the species epithet *E. australiensis*. Firstly, analysis of the genome of *E. australiensis* MST-111070 with the TYGS [39] (first time on 28 December 2020) revealed that its closest relatives are *E. hyalina* NBRC 13850T and *E. scabrispora* DSM 41855 [digital DNA hybridization (dDDH d4) scores of 54.8 and 30.9%, respectively]. TYGS analysis also highlighted the close relationship between the *Embleya* and *Yinghuangia* genera (Fig. S5). As the TYGS database contains only genomes of type strains, and it is not constantly updated, we performed an extended analysis adding in any genomes identified in NCBI databases as being possibly related to strain MST-111070 using both TYGS and GGDC [39,54]. This gave additional reassurance that this strain is a member of the genus *Embleya*, which is closely related to *Yinghuangia* (Fig. S6). However, there were some discrepancies between the methods, most notably that in the extended 16S analysis, *Yinghuangia* was paraphyletic (Fig. S6b). Additionally, *Parastreptomyces abscessus* clades within *Yinghuangia* and *Streptomyces lasii* clades within *Embleya*. To preserve the monophyly of *Embleya*, *Streptomyces* and *Yinghuangia*, these strains should be reassigned to *Yinghuangia* and *Embleya*, respectively.

**Fig. 2.**
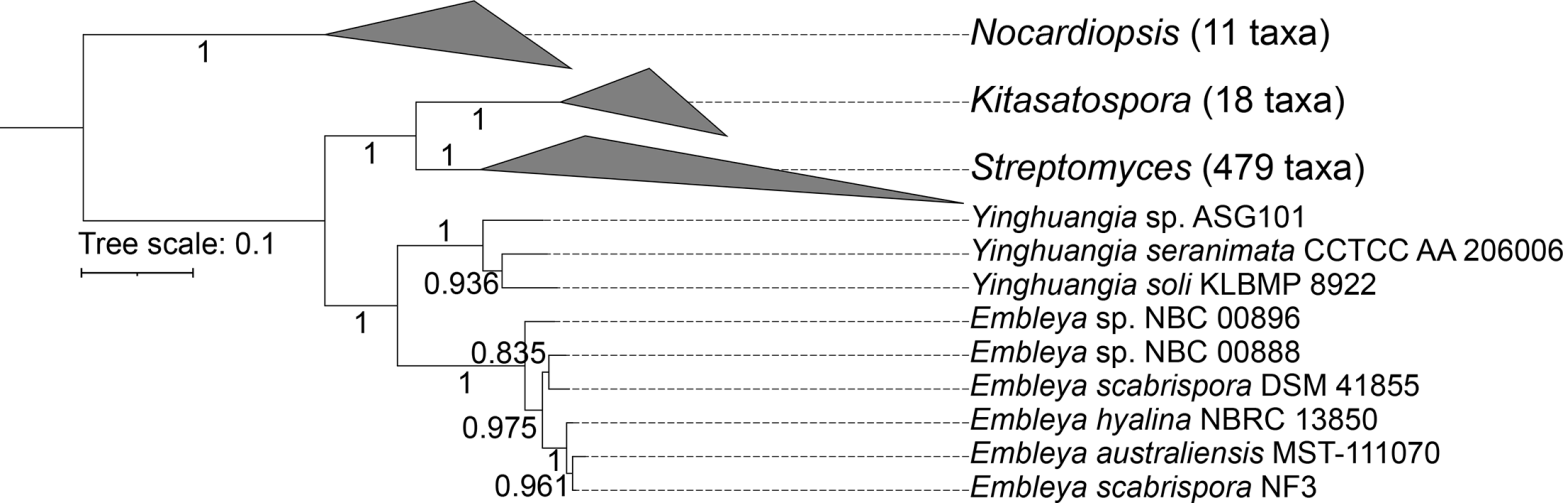
Phylogenetic tree of *Streptomycetaceae*. A phylogenetic tree of 515 strains inferred from 11 loci (Table S1) by getphylo [41] using Fasttree2 [81] and visualized in iTOL [42]. Clades for *Nocardiopsis* (outgroup), *Kitosatospora* and *Streptomyces* have been collapsed to highlight the inter-generic relationships, especially between *Yinghuangia* and *Embleya*. The tree shows that each genus is monophyletic with maximum support. Branch supports are approximate maximum likelihoods.

To clarify the phylogenetic relationship within the *Streptomycetaceae* family and other Actinobacteria genera, we undertook an independent and extensive analysis with getphylo [41] to build a genome-scale phylogenetic tree from 515 complete actinomycete genomes (Fig. 2, Table S1). This phylogeny supports the placement of *E. australiensis* MST-111070 within the genus *Embleya* and supports *Embleya* as a monophyletic group within the *Streptomycetaceae* family, among strains with high-quality sequenced genomes. It also confirms *Yinghuangia* as a monophyletic sister genus.

### EEC1: secondary chromosome, chromid or megaplasmid?

There has been much discussion over the distinct types of bacterial replicons [2,15]. Bacterial genomes are typified by a singular linear or circular chromosome. Many bacteria have additional replicons that are typically termed plasmids or megaplasmids. The distinction between plasmids and megaplasmids is solely based on size. Generally, a plasmid is considered a megaplasmid if it is >350 kb in size [2,15]. Plasmids and megaplasmids are ‘non-essential’ replicons that differ from the chromosome in function and composition. A third type of replicon, the secondary chromosome has been described in specific bacterial lineages, most notably proteobacteria (*Agrobacterium* [55], *Burkholderia* [56,57], *Pseudoalteromonas* [16,58], *Rhodobacter* [59] and *Vibrio* [60]). An alternative name, ‘chromid’, was proposed by Harrison *et al.* [15]. Chromids share features of both plasmids and chromosomes; ‘not a chromosome, not a plasmid’. Most importantly, they replicate using a plasmid-like mechanism. It is hypothesized that chromids have evolved from plasmids but, over long evolutionary time frames, have become essential through the transfer of core genes from the chromosome [15]. Chromids, therefore, demonstrate conservation on a higher taxonomic order and are typically conserved across the genus. An additional distinction has been proposed in which the term ‘secondary chromosome’ is reserved for replicons that have arisen through a schism of the chromosome [2]. However, for reasons outlined below, this distinction is problematic, and, in most fields, the term secondary chromosome is used synonymously with chromid [56,61, 62]. For clarity, the features of plasmids, chromids and chromosomes are listed in Table 1.

#### Replicon size

The first and most apparent feature of any replicon is its size. The chromosome is universally the largest replicon in the bacterial genome. Chromids, when present, are the second largest, i.e. larger than any plasmid. EEC1 is 14 times larger than the next biggest plasmid, EEC2. EEC1 is nearly 60% the size of the chromosome and accounts for 35.5% of the total genome. This clear distinction in size makes EEC1 larger than the typically agreed size for megaplasmids [2,15] (<2 Mb) and into the realm of chromids and chromosomes.

#### Nucleotide composition and codon usage

**Fig. 3.**
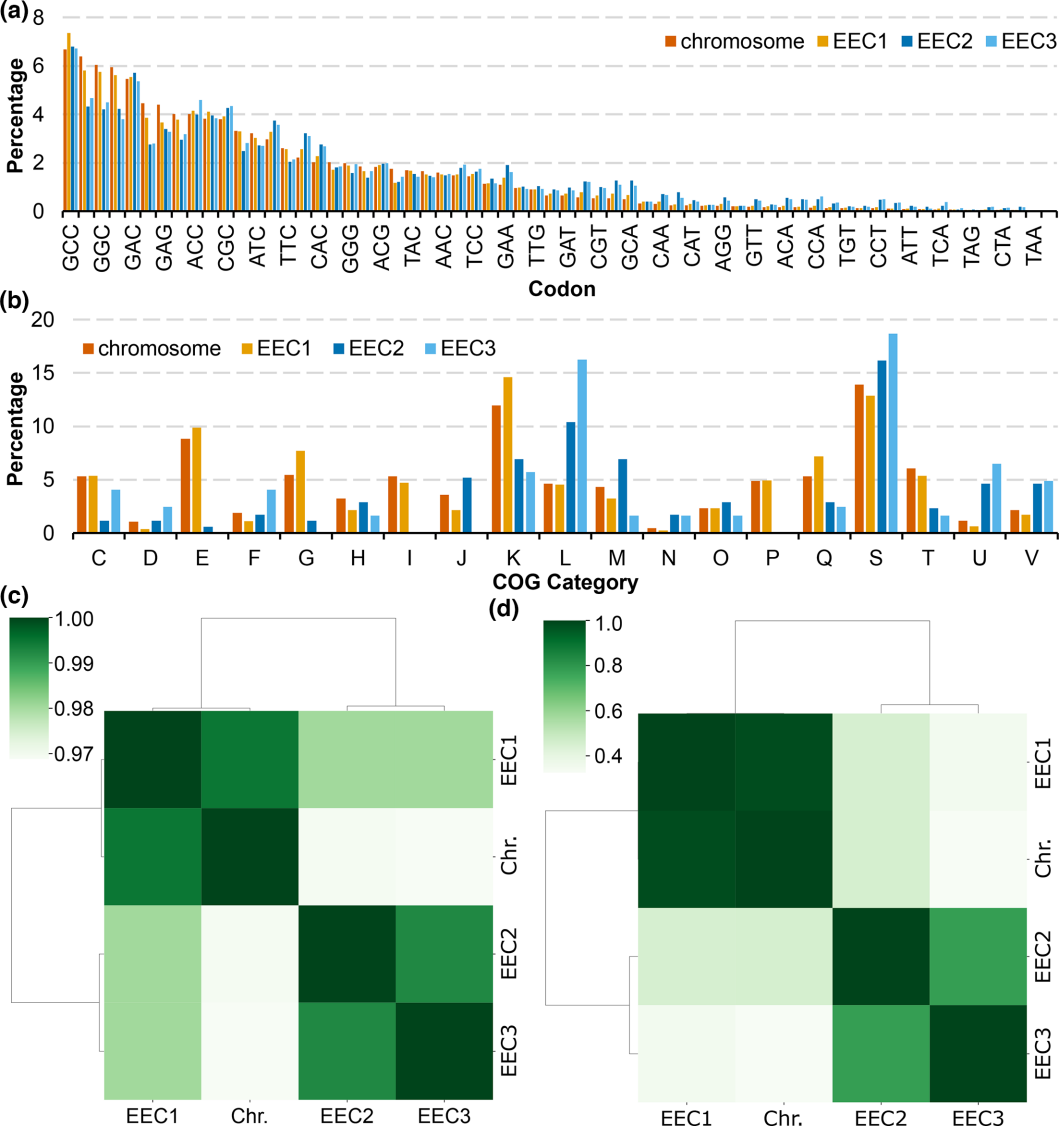
Biochemical and functional composition of *E. australiensis* MST-111070 replicons. Bar charts show the percentage abundance of (**a**) codons and (**b**) COG categories with at minimum 1% abundance across the chromosome and the three extrachromosomal elements (EEC1–EEC3) (C: energy production and conversion; D: cell cycle control, cell division, chromosome partitioning; E: amino acid metabolism and transport; F: nucleotide transport and metabolism; G: carbohydrate metabolism and transport; H: coenzyme transport and metabolism; I: lipid metabolism; J: translation, ribosomal structure and biogenesis; K: transcription; L: replication and repair; M: cell wall/membrane/envelope biosynthesis; N: cell motility; O: post-translational modification, protein turnover, chaperones; P: inorganic ion transport and metabolism; Q: secondary metabolites biosynthesis, transport and catabolism; S: function unknown; T: signal transduction; U: intracellular trafficking and secretion; and V: defence mechanisms). Heatmaps show the Spearman’s rank correlation for (**c**) the codon usage (calculated with codoniser, this study) and (**d**) the COG category abundance (calculated with egger, this study).

Chromids share similar G+C content and codon usage with the chromosome, whereas plasmids typically have different G+C content (lower in the case of *Streptomycetaceae*)[2,15]. We clearly observe this pattern in the genome of *E. australiensis* MST-111070. The chromosome and EEC1 share almost identical G+C contents of ~71.6 mol% compared to 69.1 and 69.5 mol% for EEC2 and EEC3, respectively. The codon usage in EEC1 is also very close to that of the chromosome. In addition, the chromosome and EEC1 show a greater degree of codon bias than the plasmids, which are more likely to utilize rare codons (Figs 3 and S7).

#### Replicon function and the presence of core genes

A defining feature of plasmids is that they are non-essential. Meanwhile, chromids contain core genes and are believed to be essential. Analysis of the genome with the BUSCO [36] pipeline revealed that EEC1 contained a significant number of BUSCOs duplicated with the chromosome (Table 3). However, EEC1 also carried 27 unique orthologues that are universally present in *Streptomyces* spp., suggesting that it may be essential. In addition, analysis of orthologous proteins with the European Galaxy server [38] identified a total of 4,415 reciprocal best hits between the proteins encoded in the chromosome of *Streptomyces coelicolor* A3(2) (7,846 proteins from NCBI annotation of accession AL645882.2) and proteins encoded in *E. australiensis* MST-111070 chromosome plus EEC1. Of these hits, 3,267 corresponded to the chromosome of *E. australiensis* MST-111070 (54% of 6,009 proteins from NCBI annotation of accession CP182422.1) and 1,148 corresponded to proteins encoded by EEC1 (31% of 3,685 proteins from NCBI annotation of accession CP182423.1). The conservation of EEC1 orthologues across genera provides additional validation that EEC1 encodes genes central to the biology of the strain.

**Table 3.** Comparative analysis for assembly completeness using BUSCO The number of BUSCOs indicated for each database corresponds to running the pipeline with the full assembly (bold) versus the assembly lacking the 4.2 Mb EEC1. Results indicate that EEC1 carries several duplicated BUSCOs, but also that without this replicon, the genome is missing 27 genes typically present in *Streptomycetales* (*Kitasatosporales*) species.

	Streptomycetales	Actinobacteria_class	Actinobacteria_phylum	Bacteria
Complete BUSCOs	**1,453**/1,427	**356**/356	**292**/292	**124**/124
Complete and single-copy BUSCOs	**1,427**/1,412	**348**/351	**286**/288	**120**/122
Complete and duplicated BUSCOs	**26**/15	**8**/5	**6**/4	**4**/2
Fragmented BUSCOs	**11**/10	**0**/0	**0**/0	**0**/0
Missing BUSCOs	**115**/142	**0**/0	**0**/0	**0**/0
Total BUSCO groups searched	**1,579**/1,579	**356**/356	**292**/292	**124**/124

We also compared functional content of the replicons by comparing the frequency of functionally annotated COGs). We annotated the *E. australiensis* MST-111070 genome using eggNOG [43] as implemented in eggNOG-mapper [44]. The analysis revealed that EEC1 is functionally more similar to the chromosome than the plasmids, whereas the plasmids are more alike (Fig. 3). The chromosome and EEC1 are enriched in metabolic functions (E, G, I, P, Q) and functions associated with transcription (K) and signal transduction (T). On the other hand, the two smaller replicons are enriched in genes for replication and repair (L), intracellular trafficking, secretion and vesicular transport (U) and defence (V). They also contain a much higher proportion of genes with unknown or unassigned functions. This disproportion may be due to size, since smaller replicons will dedicate a proportionally higher percentage of genes to maintenance and replication. However, the complete lack of some COGs from the plasmids, namely E (amino acid transport and metabolism), I (lipid transport and metabolism) and P (inorganic ion transport and metabolism), shows a definitive functional bias. These COGs are each associated with primary metabolic functions and suggest that the EEC1 has a much more central role in metabolism than either of the plasmids.

Finally, EEC1 is enriched in BGCs. BGCs are co-located groups of genes responsible for the biosynthesis of small molecules with specialized functions such as antibiotics and siderophores [32,63]. Analysis of the *E. australiensis* MST-111070 genome with antiSMASH 7.0 [32] predicted a total of 49 biosynthetic regions (Table S3, Fig. S9). Surprisingly, the majority of these regions (25) are found on EEC1 and not the chromosome (24). The plasmids contain a single BGC each. For its size, EEC1 shows a remarkable density of specialized biosynthetic machinery, almost double that of the chromosome. Although the majority of the BGCs encode the biosynthesis of unknown products (cryptic pathways), there are several BGCs that can be assigned to molecules known to be produced by this strain (Table 4). Antibiotic L-156602 was assigned to a hybrid polyketide-non-ribosomal peptide synthase BGC due to the domain organization of the modular megasynthases and its similarity to the BGC of the related acylated cyclic hexadepsipeptide polyoxypeptin [64]. Nybomycin was assigned to a small BGC with high similarity to the nybomycin BGC previously described [23]. Finally, leptomycins and kazusamycin were assigned to a polyketide BGC that showed high nucleotide identity (92%) and identical organization to the BGC described in patent US7288396B2 [65]. The biosynthesis of the known products of *E. australiensis* MST-111070 is restricted to the chromosome (nybomycin and leptomycins and L-156602), although the biological significance of this observation, if any, is unclear.

**Table 4.** Selected BGCs from a curated antiSMASH output for the genome of *E. australiensis* MST-111070

BGC*	Type†	From nt‡	To nt‡	Most similar known BGC§
Chr-7	NRPS, T1PKS, indole	980,466	1,101,259	**Antibiotic L-156602**¶
Chr-19	NRPS-like, betalactone	5,940,563	5,984,102	**Nybomycin (BGC0001965)**
Chr-24	T1PKS	6,905,975	7,081,035	Leptomycin/kazusamycin**
EEC1-12	Lanthipeptide-class-iii	1,828,683	1,851,442	SapB homologue††
EEC1-19	T2PKS	2,792,990	2,865,499	Spore pigment (BGC0000271)‡‡

* Chr denotes the chromosome.

† BGC type as annotated by antiSMASH.

‡ Start and end positions of the the BGC as estimated by antiSMASH.

§ Curated from antiSMASH predictions and manual analysis; in bold type, compounds empirically identified as produced by *E. australiensis* MST-111070 (by Microbial Screening Technologies Pty. Ltd., data not shown); underlined, BGCs highly conserved and essential for the life cycle of streptomycetes.

¶ Assigned based upon similarity to the known polyoxypeptin BGC (BGC0001036).

** Identified by blastn search and PKS similarity: 92% identity with Fig. 5 from patent US7,288,396 B2.

†† Assigned by antiSMASH as most similar to SAL-2242 (BGC0000546) and SapB (BGC0000551) from *S. coelicolor* A3(2).

‡‡ Highly conserved BGC among streptomycetes.

Strikingly, among the BGCs encoded by EEC1 are both the BGC required for biosynthesis of the spore pigment and a SapB homologue. Both BGCs play an important role in the morphological development and life cycle of *Streptomyces* spp. and related Actinobacteria: spore pigment is found in mature spores, whereas SapB-like peptides play a vital role in the development of aerial mycelium [66,67]. Furthermore, NCBI Prokaryotic Genome Annotation Pipeline [68] annotated two copies of the Tap-Tpg encoding genes, required for replication of the linear replicons in streptomycetes [13,69], all of them in the EEC1 replicon (ACMXN5_30930–ACMXN5_30935 and ACMXN5_34795–ACMXN5_34800 locus tags in sequence with accession CP182423) with amino acid identity between 43 and 55% with *S. coelicolor* A3(2) Tap-Tpg pair (genes SCO7733 and SCO7734 of sequence with accession AL645882). Again, the presence of these BGCs on EEC1 supports the conclusion that it encodes essential cellular functions.

#### Replicative mechanism

**Fig. 4.**
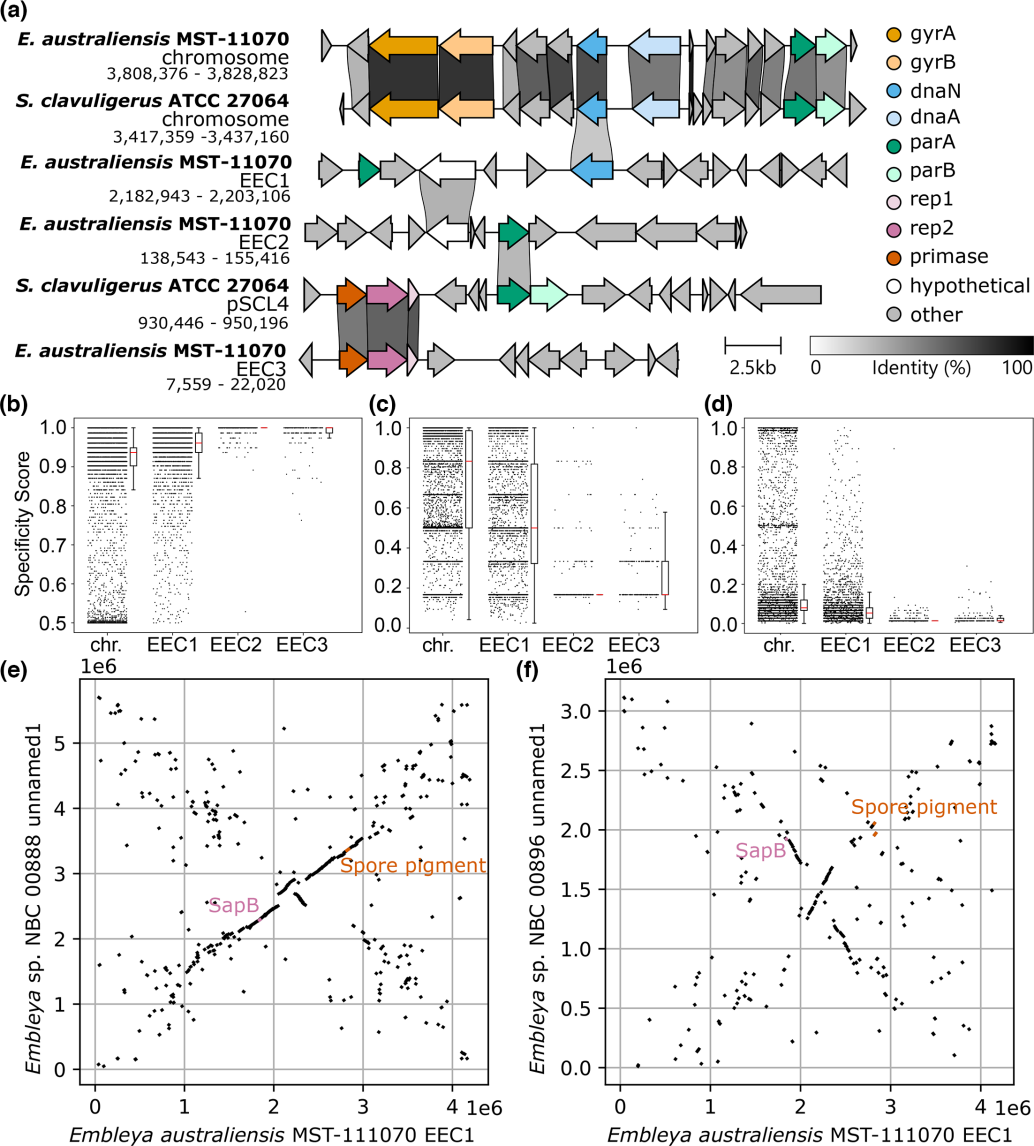
Comparison of the *E. australiensis* MST-111070 chromosome (chr.) and its extrachromosomal elements (EEC1–EEC3). A comparison of predicted replicon regions carrying (a) the origin of replication, modified from clinker output [82]. Links show genes encoding proteins sharing an identity of >30%. Genes are coloured by the predicted function based on profile HMMs [83]. Strip plots showing the specificity scores of every encoded protein at the (b) strain-, (c) genus- and (d) family-level. Additional box plots are provided to show the median (red) and the quartile spread of the data points. (e) and (f) Dot plots showing the similarity and synteny of the secondary replicon EEC1 from *E. australiensis* MST-111070 with large secondary replicons identified in high-quality genome assemblies from two additional *Embleya* species.

Despite their overall similarity to the chromosome, chromids utilize plasmid-type replication systems [2,15]. In *Streptomyces* species, linear plasmids commonly replicate bidirectionally from a central replicon consisting of a DNA helicase (*rep2*) and its corresponding iteron (*rep1*) [70,72]. In contrast, the chromosomal origin of replication is flanked by the gene encoding the replication initiation protein, *dnaA*, and the gene encoding the DNA polymerase subunit, *dnaN* [73,75]. Both replication systems require a set of partitioning genes *parA* and *parB* [5,76, 77]. The origin of each *E. australiensis* MST-111070 replicon was identified using Diamond homology searches against previously described replicons [5,77] (Table S2, Fig. 4a). The chromosomal origin was easily identified by the presence of *dnaA* and *dnaN* homologues in addition to homologues of *parA*, *parB*, *gyrA*, *gyrB* in the local regions (loci: ACMXN5_16320–ACMXN5_16385). The origin of EEC3 was also easily identified with homology searching due to the presence of close homologues of *rep1* and *rep2* (ACMXN5_50245 and ACMXN5_50250) and *parA* and *parB* (ACMXN5_50205 and ACMXN5_50210). The replication systems of EEC1 and EEC2 are much more difficult to define. EEC1 lacks the canonical plasmid replication system, but it also lacks genes required for chromosomal replication. It carries a copy of *dnaN* (ACMXN5_39850) but is lacking a corresponding *dnaA* locus. A distant homologue of *parA* is also present in the vicinity (ACMXN5_39820). Further searching using PFAM HMMs also failed to find *parB* and *dnaA* homologues. One possibility is that the replication of EEC1 relies on machinery encoded in the chromosome, i.e. it cannot replicate independently from the chromosome; however, given that we were unable to find *parS* sites similar to those in the chromosome (GTTTCACGTGAAAC) [78], this seems unlikely. EEC1 may also replicate through an, as yet, unidentified mechanism. EEC2 has both *parA* and *parB* homologues (ACMXN5_49550 and ACMXN5_48975); however, they are not clustered. Additionally, although EEC2 encodes several helicases, none show homology with helicases from canonical plasmid or chromosomal replicons. Overall, these results highlight the diversity of replicons in *Streptomycetaceae* that have yet to be characterized.

GC skew analysis was consistent with the location of the chromosomal origin and supported that EEC3 is a circular plasmid (Fig. S10). The GC skew of EEC1 shows a plateau between 1.9 and 2.8 Mb, which may indicate a recent rearrangement (Fig. S10).

#### Evolutionary origin and genus-specificity

Another hallmark feature of chromids is that they contain a high number of genus-specific genes (i.e. genes that only have close homologues within the genus) [15]. We thus estimated the specificity of genes on the strain-, genus- and family-level (Fig. 4b–d). In general, the chromosome shows a trend of increasing specificity with higher taxonomic rank (i.e. lower proportion of strain-specific genes), whereas the plasmids EEC2 and EEC3 show the inverse (higher proportion of strain-specific genes). At all taxonomic ranks, EEC1 shows similar specificity to the chromosome, exhibiting highest levels of specificity at the strain- and genus-level. While most of the genes on EEC2 and EEC3 are unique to those plasmids, EEC1 encodes genes that are also encoded by other *Embleya* strains. This indicates a high level of conservation of EEC1 across *Embleya* species, in line with the chromosome.

While preparing this manuscript, high-quality genome assemblies for two additional *Embleya* species were published from the New Bioactive Compounds (NBC) collection [12]. These genome assemblies also contain large secondary replicons (3.1 and 5.7 Mb) that exhibit similar traits to EEC1 and share large regions of homology and synteny (Fig. 4e, f), with a quarter of proteins encoded by EEC1 (24.2%) conserved across the secondary replicons of all three species (>70% identity). Sixteen proteins were conserved with an identity above 95% across all three replicons (Table S4). These conserved genes include *parA* and a hypothetical protein close to the replicon and two clusters of genes involved in carbohydrate metabolism, transport and regulation. Given the genetic distance of these strains, the conservation of these genes is especially notable and points to EEC1’s remarkable conservation across the genus.

The conserved regions also include the BGC for spore pigment and, like *E. australiensis* MST-111070, the secondary replicon of *Embleya* sp. NBC 00888 also carries a BGC homologous to SapB (accession NZ_CP108785.1) (Figs 4e and S11). All replicons also share other homologous BGCs that encode the biosynthesis of specialized metabolites, most notably a homologue BGC to the siderophore peucechelin and other lanthipeptide BGCs (Fig. S11). Prior to the publication of the NBC strains, five other genome assemblies of *Embleya* strains were available in the public databases [three classified as *Embleya* spp. (*E. hyalina* NBRC 13850 GCA_003967355.1_ASM396735v1, *E. scabrispora* KM4927 GCA_000372745.1_ASM37274v1 and *E. scabrispora* NF3 GCA_002024165.1_SSNF3.0) and two wrongly classified as *Streptomyces* sp. (*Streptomyces* sp. SID3343 GCA_009865215.1_ASM986521v1 and *Streptomyces* sp. SID5474 GCA_009862895.1_ASM986289v1)]. These are all highly fragmented assemblies and difficult to use for analysis, apart from that of strain *E. scabrispora* NF3, whose assembly contains only eight contigs, with one contig of over 7 Mb likely representing the nearly complete chromosome, and which does not carry the BGC for the spore pigment (instead, the BGC for the spore pigment appears in a 1,126 kb contig highly similar to EEC1). The *E. scabrispora* NF3 assembly also shows another 1,248 kb contig with high similarity to another part of EEC1 (Fig. S12) that carries BGCs for several lanthipeptides and a siderophore most similar to peuchechelin, just as EEC1 does (Fig. S12). In all cases, genome alignment of *E. australiensis* MST-111070 with these five assemblies using Mauve [35] indicates that many of the contigs of the incomplete genomes map to EEC1, providing further evidence of this replicon as a characteristic of the *Embleya* genus (Fig. S13).

Also noteworthy is the fact that the *Streptomycetaceae* genus closest to *Embleya* is the rare genus *Yinghuangia*. The complete genome of *Yinghuangia* sp. ASG 101 (NCBI accession ASM2116573v1) is available. It exhibits a single replicon, the chromosome, with typical streptomycete characteristics, including the BGC for the spore pigment.

### Proposal of EEC1 as a secondary chromosome that is characteristic of the *Embleya* genus

The 4.2 Mb linear replicon EEC1 from *E. australiensis* MST-111070 is the largest secondary replicon described in bacteria, thus far. It is typified by a composition similar to the chromosome in terms of G+C content, codon usage and overall function and shares the same TIRs. EEC1 differs from the chromosome in that it contains fewer family-specific genes and is enriched in BGCs. This unique character of EEC1 makes it difficult to characterize under conventional definitions.

According to the definition introduced by Harrison *et al.* [15], chromids: (i) utilize plasmid-like replication and maintenance systems; (ii) have chromosome-like nucleotide composition and (iii) carry core genes (i.e. those that are found on the chromosome of other species). Additionally, chromids are conserved across the genus and carry a large proportion of genus-specific genes. In many respects, EEC1 resembles a chromid due to its chromosome-like nucleotide composition and codon usage and its genus-level distribution. However, unlike chromids, it does not appear to utilize an established plasmid-like replicon. EEC1 appears to contain an incomplete chromosomal replicon, including highly conserved homologues of ParA (>95% identity across *Embleya* spp.) and DnaN. It also has identical TIRs to the chromosome, suggesting that its linear integrity is maintained through the same mechanism. This means that EEC1 does not meet the criteria of either a chromid or a plasmid.

Other replicons from the related genus *Streptomyces* also defy classification under this system. Most notably, pSCL4 from *S. clavuligerus* ATCC 27064 [5]. At 1.8 Mb, this is among the largest secondary replicons from *Streptomyces* described to date. Analysis of pSCL4 under the framework provided in this study indicates that it is similar to a chromid due to its chromosomal properties (Table S5, Figs S7 and S8). pSCL4 encodes functionally similar genes to the chromosome, encodes genes shown to be essential for chromosome stability [13] (*tap* and *tpg*) and has codon usage similar to the chromosome. Additionally, it harbours a replication system similar to the megaplasmid SCP1. Unlike EEC1 and various chromids, it is not distributed across the genus; rather, it is found uniquely in *S. clavuligerus* strains. Additionally, mutation of pSCL4 *parB* resulted in plasmid curing, demonstrating that pSCL4 is not strictly essential to the survival of the strain (but is essential for the maintenance of the linear topology of the chromosome) [13]. We analysed additional large *Streptomyces* replicons, all of which are strain specific and encode an SCP1-like replicon (Table S6). One interesting example from elsewhere in the *Streptomycetaceae* family is pSCATT (1.8 Mb) from *Streptantibioticus cattleyicolor* NRRL 8057 that harbours a unique origin of replication and is maintained with a copy number of 1 per chromosome [4]. However, as *St. cattleyicolor* is the only representative of the genus with a complete genome sequence, it is impossible to comment on the conservation of pSCATT and its classification.

Another important aspect is the evolutionary origin of the replicon. There are two main hypotheses for the origin of secondary replicons: the schism hypothesis, whereby the replicon is formed by a split in the chromosome, and the plasmid hypothesis, whereby the replicon originates as a plasmid and attains chromosomal character over time. DiCenzo *et al.* took this idea further and used it as a basis for the classification of chromids and secondary chromosomes [2]. Using this approach, there is evidence to categorize EEC1 as a secondary chromosome due to its high similarity to the primary chromosome and large proportion of chromosomal orthologues. However, it is incredibly difficult, if not impossible, to prove the schism hypothesis definitively. Genetic interchange between replicons is high and many recombinational events have been observed, such as hybridization between secondary replicons and the chromosome [79,80] and the interchange of terminal repeats [46] (see also this study). Given that a defining feature of chromids is that they are compositionally indistinguishable from the chromosome, it is unclear what burden of evidence would be required to definitively claim that a replicon was formed by a schism event, as opposed to a series of recombinatorial events, that would not be lost over such long evolutionary timescales. Due to this quirk in the nomenclature, all previously described secondary chromosomes are also chromids; the terms have become synonymous and are used largely interchangeably in the literature [56,57, 62, 80]. Ultimately, EEC1 shares the characteristics of previously described secondary chromosomes but does not have a confirmed plasmid-type replicon. Therefore, we propose EEC1 as a secondary chromosome but not a chromid.

EEC1 is unique and highlights the difficulty of assigning arbitrary categories to biological entities. As with any system of classification, there will always be exceptions that fall outside of the standard definitions. EEC1 and pSCL4 are two distinct examples that fall outside the established categories of chromids and megaplasmids. The study of genomic structure and secondary replicons is a developing field, particularly in *Streptomycetaceae*. While the current framework of classification provides valuable structure, it is important to recognize that secondary replicons appear to exist on a spectrum of decreasing chromosomal character and increasing evolutionary novelty.

## Conclusion

Here, we describe the genome of *E. australiensis* MST-111070 and its extraordinary 4.2 Mb secondary chromosome, EEC1. To our knowledge, EEC1 is the largest bacterial secondary chromosome described to date and the first secondary chromosome to be identified in an actinobacterial strain. Unlike other secondary chromosomes (chromids), EEC1 does not maintain itself through a known plasmid-like replication system. Instead, it appears to replicate through a novel mechanism with similarities to the chromosomal replicon, suggesting it may have arisen through a separate evolutionary mechanism to other secondary chromosomes. Most strikingly, the presence of similar secondary chromosomes for all other *Embleya* species identifies it as an essential part of the evolutionary history of the genus and a distinguishing characteristic amongst the *Streptomycetaceae*. These observations distinguish EEC1 from previously described secondary replicons and we conclude that EEC1 should be considered a secondary chromosome, but not a chromid. This study underscores the importance of optical mapping in confirming the results of genome sequencing and we hope the discovery of EEC1 will encourage further in-depth characterization of extraordinary bacterial replicons.

## Supplementary material

10.1099/mgen.0.001704 Supplementary Material 1.

## Abbreviations

BGCs: biosynthetic gene clusters
BUSCO: benchmarking universal single-copy orthologs
COG: clusters of orthologous gene
EEC: *Embleya* extrachromosomal element
NBC: new bioactive compounds
PyPI: Python package index
TIRs: terminal inverted repeats
TYGS: type strain genome server
