# Supplementary material for: Evidence supporting the first secondary chromosome in Actinobacteria as a hallmark of the Embleya genus
Source: Microb Genom. 2026 May 7;12(5):001704. doi: 10.1099/mgen.0.001704 (PMC13152386; doi:10.1099/mgen.0.001704)
Supplement: Supplementary Material 1. [file mgen-12-01704-s001.pdf]

SUPPORTING INFORMATION for

Evidence supporting the first secondary chromosome in actinobacteria as a hallmark of the *Embleya* genus

J. P. Gomez-Escribano, S. Dorai-Raj, D. Baker, E. Lacey, B. Wilkinson and T. J. Booth

Contents

1. EXTENDED METHODS DESCRIPTION 2
Sequence data analysis 2
Bionano data analysis 2

2. SUPPLEMENTARY TABLES 5
Table S1. Loci selected for phylogenetic analysis by getphylo. 5
Table S2. Identification of genes associated with chromosomal or plasmid origins of replication. 5
Table S3. Curated antiSMASH output for the genome of *Embleya australiensis* MST-111070 6
Table S4: Highly conserved proteins between EEC1-like replicons. 8
Table S5: Features of *Embleya australiensis* MST-111070 EEC1 and large linear *Streptomyces* spp. replicons. 9
Table S6: Analysis of other large replicons from *Streptomycetaceae* 10
Table S7: P-values associated with codon usage correlation in *Embleya australiensis* MST-111070 (Figure 2c) 11
Table S8: P-values associated with COG function correlation (Figure 2d) 11
Table S9: P-values associated with codon usage correlation in *S. clavuligerus* ATCC 27064 (Figure S7) 11
Table S10: P-values associated with COG function correlation (Figure S8) 11

3. SUPPLEMENTARY FIGURES 12
Figure S1. Identification and analysis of terminal inverted repeats. 12
Figure S2. Alignment of Bionano molecules and DNA sequence assembly. 13
Figure S3. Alignment of Bionano and sequence genome assemblies. 14
Figure S4. Overview of the alignment of the 211 Kb EEC3 circular plasmid with its corresponding BioNano contig. 15
Figure S5. Phylogenetic analysis provided by TYGS for full genome and 16S analysis of *Embleya australiensis* MST-111070. 16
Figure S6. Extended phylogenetic analysis provided by TYGS for full genome and 16S analysis of *Embleya australiensis* MST-111070. 17
Figure S7. Heatmap and hierarchical clustering of codon usage correlation for secondary replicons in *S. clavuligerus* ATCC 27064. 20
Figure S8. Heatmap and hierarchical clustering of COG utilisation correlation for secondary replicons in *S. clavuligerus* ATCC 27064 21
Figure S9. Original output of the annotation of putative specialised metabolism biosynthetic gene clusters by antiSMASH 22
Figure S10: Cumulative GC-skew of *Embleya australiensis* MST-111070 replicons. 25
Figure S11. antiSMASH annotation of the large secondary replicon of high-quality genome assemblies of other *Embleya* species. 26
Figure S12. Artemis Comparison Tool view of the genome sequence comparison. 27
Figure S13. Alignment of the genome assemblies of *Embleya australiensis* MST-111070 with available genome assemblies from other *Embleya* strains. 27

4. REFERENCES 29

1. EXTENDED METHODS DESCRIPTION

Sequence data analysis

General visualisation, analysis, and manipulation of DNA sequence data was performed with computer programs Artemis[1], Artemis Comparison Tool[2], and NotePad++ (http://notepad-plus-plus.org/). Alignments and assembly of sequences was performed with Staden Package[3, 4]. Similarity searches were performed with BLAST+ [5] at the NCBI web server (http://www.ncbi.nlm.nih.gov/blast/), or on a standalone computer with prfectBLAST 2.0[6]. Annotation of gene function and genetic features was performed with antiSMASH (version 7 with "relaxed strictness" and all options enabled)[7] and RAST (Rapid Annotation using Subsystem Technology)[8, 9] using Prodigal[10] for CDS calling, and with frame-shift fixing, and metabolic model building options. Mauve[11] was used for full genome comparisons and contigs reordering. Busco[12] was used to analyse the presence of orthologous genes per taxonomic group (version BUSCO v5.4.4 with hmmsearch 3.1, bbtools 39.01, prodigal 2.6.3, with databases actinobacteria_class_odb10; actinobacteria_phylum_odb10; bacteria_odb10; streptomycetales_odb10). TYGS[13] and GGDC[14, 15] were used for genome-based and 16S rDNA-based taxonomical studies (with Extended Maximum Likelihood and Maximum Parsimony 16S rRNA analysis, which includes type strains for which the genome sequence is not available).

Bionano data analysis

The BNX files with Bionano molecules information were processed with Bionano's software IrysView Genomic Analysis Viewer, version 2.5.1.29842, with versions r5134 of PipelineCL.py, r5122 of RefAligner.cpp, r5122 of Assembler.cpp, and r5146 of Hybrid Scaffold; all running on Microsoft Windows 10 64 bits with Python 2.7.8. IrysView was used for alignment of Bionano molecules over the consensus genome sequence and visualisation, following the guidelines in the Bionano document "IrysView® v2.5.1 Software Training Guide. Document Number: 30035 Document Revision: G, 2016"; the result of Molecule Quality Report (alignment over reference sequence) was interpreted according to the document "Guidelines for Interpreting the Bionano Molecule Quality Report. Document number30175, Rev A" and following advice from BioNano Technical Support.

During import of BNX files into IrysView, molecules were filtered for a minimum of 100 kb length with Dynamic Threshold label SNR.

Data for all scans were merged with default options, resulting in a total of 229509 molecules accounting for 37288.4 Mb, with a molecule N50 of 158.5 Kb.

The following figure shows an overview of the general pipeline for Bionano Optical Mapping data collection and analysis. **1**. High-molecular weight genomic DNA (i.e. careful extraction as to minimise fragmentation of the chromosome and large extrachromosomal replicons) is labelled with a fluorophore at positions where a specific sequence motif occurs. **2**. The labelled DNA fragments are injected into the Bionano chip, where they are separated, extended, and individual DNA molecules are imaged to high-resolution, generating a contiguous linear distribution map of the specific sequence motif along the DNA fragments, what is called "bionano molecule". **3**. The bionano molecule maps are assembled with Bionano's provided software to, **4**, create a contiguous map of the distribution of sequence motifs along entire replicons (*de novo* assembly of Bionano molecules). This map can be used to, **5**, assess the correctness of a full genome sequence assembly by matching the observed fluorescence labels distribution to that predicted from the genome sequence (alignment of BioNano *de novo* assembly to DNA-sequence assembly). This can also be done by mapping the "bionano molecules" directly to the sequence assembly (Molecules-to-Reference Alignment, **6**). Full details are given next.

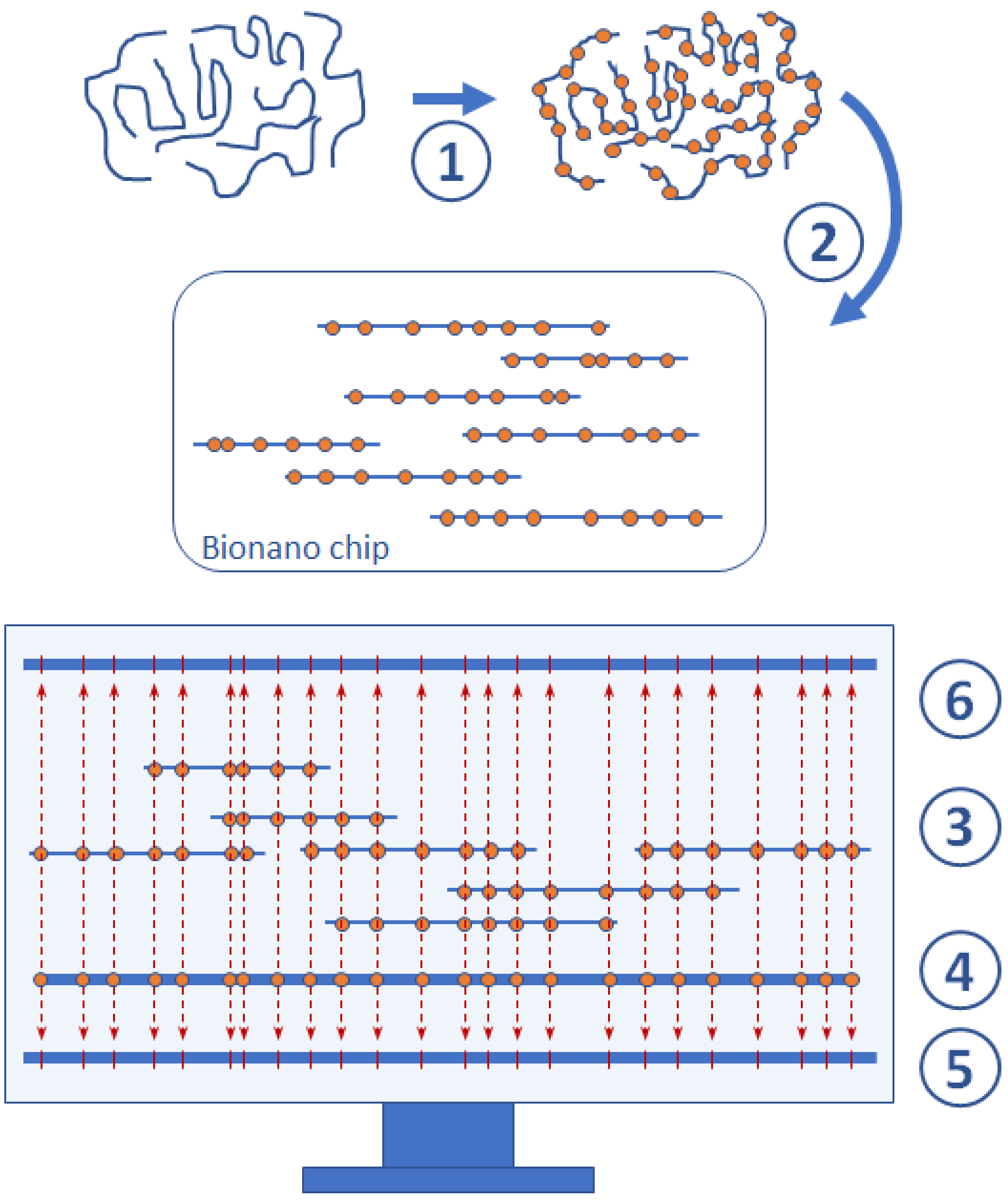

Molecules-to-Reference Alignment

Molecule Quality Report (MQR) aligns the Bionano molecules to a reference sequence, being a good indicator of the accuracy of the DNA-sequence assembly. MQR was performed without molecule subsampling, 3 iterations, and the following molecule-to-reference RefAligner options:

-nosplit 2 -BestRef 1 -biaswt 0 -Mfast 0 -FP 1.5 -FN 0.15 -sf 0.2 -sd 0.0 -A 5 -outlier 1e-3 -outlierMax 40 -endoutlier 1e-4 -S -1000 -sr 0.03 -se 0.2 -MaxSF 0.25 -MaxSE 0.5 -resbias 4 64 -maxmem 64 -M 3 3 -minlen 150 -T 1e-8 -maxthreads 32 -hashgen 5 3 2.4 1.5 0.05 5.0 1 1 3 -hash -hashdelta 10 -hashoffset 1 -hashmaxmem 64 -insertThreads 4 -maptype 0 -PVres 2 -PVendoutlier -AlignRes 2.0 -rres 0.9 -resEstimate -ScanScaling 2 -RepeatMask 5 0.01 -RepeatRec 0.7 0.6 1.4 -maxEnd 50 -usecolor 1 -stdout –stderr

The only parameter changed in relation with the default options was the T option (the P-value cut-off) from 1e-11 (set for human genome) to 1e-8; this is still one order of magnitude more stringent than the suggested for small genomes, 1e-7 for *Escherichia coli* and 5 Mbp genome size (see

Bionano's documentation "Guidelines for Interpreting the Bionano Molecule Quality Report. Document number30175, Rev A"

As reference DNA sequence we used version 4 of the assembly (PacBio HGAP.3, TIRs extended with Illumina, putative circular plasmid sequence starting with *parB* stop codon).

Despite the Map Rate was 42.7% (Molecules showing high similarity to reference as percentage of 'N Molecules') which is below the expected range (60-80%), the relevant statistic indicators for the quality of mapping were well within the range established by Bionano, at FP 0.48/100kbp, FP 4.4%, and FN 18.9% (limits stated in the Guidelines are FP (/100kbp) < 1.7; FP (%) < 15%; FN (%) < 21%; "FP (/100kb)" means density of molecule labels absent in the reference map (relative to reference labels); "FP (%)" means percentage of molecule labels absent in the reference map (relative to reference labels); "FN (%)" means percentage of reference labels absent in the aligned molecules (relative to reference labels); see Bionano's document number 30175 for details)

Therefore, the statistics alone indicate that the Bionano data align well with the reference sequence, supporting the correctness of our assembly version 4. Beyond the mere statistics, detailed study of the alignment reveals the excellent distribution of Bionano molecules along the DNA assembly (see Figure S2).

De novo **assembly of Bionano molecules**

Assembly was performed with the merged filtered data as before, using the settings for small size genomes (OptArgs: optArguments_small) and without reference.

The result was 8 contigs spanning a total of 14.359 Mb, with N50 of 4.33 Mb and an average coverage of 106.3 molecules:

Contig	Map ID	Size
1	1	4.330 Mb
2	2	4.363 Mb
3	4	2.022 Mb
4	11	556.986 kb
5	17	578.444 kb
6	125	551.393 kb
7	231	1.614 Mb
8	1207	343.984 kb

Alignment of BioNano De novo assembly to DNA-sequence assembly

The de-novo assembled bionano contigs were mapped and aligned to the DNA sequence assembly version 4 provided as reference, with the following options:

-maxthreads 32 -output-veto-filter _intervals.txt$ -res 2.9 -FP 0.6 -FN 0.06 -sf 0.20 -sd 0.0 -sr 0.01 -extend 1 -outlier 0.0001 -endoutlier 0.001 -PVendoutlier -deltaX 12 -deltaY 12 -xmapchim 12 -hashgen 5 7 2.4 1.5 0.05 5.0 1 1 1 -hash -hashdelta 50 -mres 1e-3 -hashMultiMatch 100 -insertThreads 4 -nosplit 2 -biaswt 0 -T 1e-8 -S -1000 -indel -PVres 2 -rres 0.9 -MaxSE 0.5 -HSDrange 1.0 -outlierBC -xmapUnique 12 -AlignRes 2. -outlierExtend 12 24 -Kmax 12 -f -maxmem 128 -BestRef 1 -stdout -stderr

2. SUPPLEMENTARY TABLES

Table S1. Loci selected for phylogenetic analysis by getphylo.

The locus ID refers to the locus tag from the seed genome *Streptomyces* sp. LX-29.

Locus ID	Alignment Length	Informative Sites	Description
LRS74_RS08715	479	276	pseudouridine synthase B (TruB)
LRS74_RS08740	435	212	transcription termination/antitermination protein NusA
LRS74_RS10140	494	209	F0F1 ATP synthase subunit beta
LRS74_RS13285	379	197	tRNA threonylcarbamoyladenosine biosynthesis protein TsaB
LRS74_RS13530	356	127	small ribosomal subunit protein uS3
LRS74_RS13545	279	81	large ribosomal subunit protein uL2
LRS74_RS13620	243	110	50S ribosomal protein L1
LRS74_RS17975	207	104	DUF47 domain-containing protein
LRS74_RS20580	361	241	ribosome hibernation promotion factor (HPF)
LRS74_RS23160	320	191	nicotinate-nucleotide adenylyltransferase
LRS74_RS24320	315	203	zinc ribbon domain-containing protein

Table S2. Identification of genes associated with chromosomal or plasmid origins of replication.

The results show the percentage identity of hits from *Embleya australiensis* MST-111070 to corresponding genes from *Streptomyces clavuligerus* ATCC27064 from Diamond[16] BlastP searches. Blank cells indicate no hit.

replicon	Chromosome				pSCL4			
	dnaA	*dnaN*	*parA*	*parB*	*rep1*	*rep2*	*parA*	*parB*
Chromosome	55.9	75.9	82.9	57.1	-	-	34.1	30.1
EEC1	-	43.3	42.1	-	-	-	-	-
EEC2	-	-	34.6	36.9	-	-	38.9	-
EEC3	-	-	44.6	-	73.4	74.2	-	47.3

Table S3. Curated antiSMASH output for the genome of *Embleya australiensis* MST-111070.

Region/ BGC[1]	Type[2]	From nt[3]	To nt[3]	Most similar known BGC[4]
Chr-1	T1PKS,linaridin	89,951	138,901	*Legonaridin (BGC0001188)*
Chr-2	terpene	150,625	171,617	*None*
Chr-3	CDPS	171,621	192,325	*None*
Chr-4	NRPS-like,NRPS,T1PKS	195,790	343,903	*Incednine (BGC0000078)*
Chr-5	thiopeptide,LAP,T1PKS	349,551	414,513	*Paromomycin (BGC0000712)*
Chr-6	terpene	882,536	903,615	*Dudomycin A (BGC0002359)*
Chr-7	indole,NRPS,T1PKS	980,466	1,101,259	**Antibiotic L 156602[5]**
Chr-8	NRP-metallophore,NRPS	1,177,888	1,235,978	Griseobactin (BGC0000368)
Chr-9	T3PKS	1,266,421	1,307,485	Alkylresorcinol (BGC0000282)
Chr-10	other	1,336,893	1,377,618	*Himastatin (BGC0001117)*
Chr-11	hydrogen-cyanide	1,439,526	1,452,331	*Aborycin (BGC0002285)*
Chr-12	NRPS,HR-T2PKS	4,277,131	4,345,962	Ishigamide (BGC0001623)
Chr-13	NAPAA,T1PKS,hglE-KS	5,295,805	5,365,248	*Hexacosalactone A (BGC0002497)*
Chr-14	lanthipeptide-class-iv	5,425,093	5,448,404	*Labyrinthopeptin A1-3 (BGC0000519)*
Chr-15	NI-siderophore	5,479,577	5,513,301	*Schizokinen (BGC0002683)*
Chr-16	NI-siderophore	5,640,743	5,670,680	*None*
Chr-17	RiPP-like	5,701,274	5,712,086	*None*
Chr-18	terpene,betalactone	5,803,329	5,857,721	Hopene (BGC0000663)
Chr-19	NRPS-like,betalactone	5,940,563	5,984,102	**Nybomycin (BGC0001965)**
Chr-20	T1PKS	6,126,397	6,199,439	*Neoabyssomicin/Abyssomicin (BGC0001694)*
Chr-21	terpene	6,559,542	6,581,863	*Polyoxypeptin (BGC0001036)*
Chr-22	RiPP-like	6,595,549	6,606,349	*Persiamycin A (BGC0002045)*
Chr-23	terpene	6,778,221	6,799,270	*None*
Chr-24	T1PKS	6,905,975	7,081,035	**Leptomycin/Kazusamycin[6]**
EEC1-1	PKS-like	294,513	335,535	*Rustmicin (BGC0000065)*
EEC1-2	NRPS,NRPS-like	524,754	578,809	*WS9326 (BGC0001297)*
EEC1-3	NRPS,NRPS-like	608,729	676,086	Himastatin (BGC0001117)
EEC1-4	NRPS,betalactone	683,228	753,983	*Ohmyungsamycin A-B (BGC0002293)*

EEC1-5	terpene,NRPS, aminocoumarin,HR-T2PKS	971,183	1,044,988	*Acyldepsipeptide 1 (BGC0001967)*
EEC1-6	RiPP-like	1,049,383	1,061,281	*None*
EEC1-7	RRE-containing, butyrolactone	1,064,960	1,091,725	*None*
EEC1-8	NAPAA	1,336,612	1,370,679	*γ-poly-L-2,4-diaminobutyric acid (BGC0002535)*
EEC1-9	hydrogen-cyanide	1,462,193	1,475,159	*None*
EEC1-10	NRPS	1,550,546	1,593,887	*Triacsin C (BGC0001983)*
EEC1-11	lanthipeptide-class-iv	1,619,382	1,641,973	*Ulleungmycin (BGC0001814)*
EEC1-12	lanthipeptide-class-iii	1,828,683	1,851,442	SapB homologue[7]
EEC1-13	NRPS	2,014,404	2,064,860	
EEC1-14	terpene	2,106,657	2,127,820	*None*
EEC1-15	terpene	2,240,391	2,261,305	*None*
EEC1-16	NRPS,T1PKS	2,426,285	2,492,967	*ECO-0501 (BGC0002098)*
EEC1-17	NRPS,lanthipeptide-class-ii	2,579,389	2,634,742	*None*
EEC1-18	NI-siderophore,NRPS	2,641,507	2,731,521	*Peucechelin (BGC0002466)*
EEC1-19	T2PKS	2,792,990	2,865,499	Spore Pigment (BGC0000271)[8]
EEC1-20	T3PKS	2,869,003	2,910,154	*R1128 (BGC0000261)*
EEC1-21	T1PKS,HR-T2PKS, arylpolyene,NRPS-like	3,295,026	3,442,703	*Akaeolide (BGC0001199)*
EEC1-22	hglE-KS	3,522,775	3,573,744	*None*
EEC1-23	lassopeptide	3,725,618	3,748,424	*None*
EEC1-24	terpene	3,796,308	3,817,516	*Ebelactone (BGC0001580)*
EEC1-25	NRPS-like	3,890,578	3,933,670	*None*
EEC1-26	NRPS-like	4,013,519	4,071,452	*Ecumicin (BGC0001582)*
EEC2-1	lanthipeptide-class-i	120,682	145,886	*None*
EEC3-1	ranthipeptide	36,794	75,454	*Desulfoclethramycin/Clethramycin (BGC0002498)*

[1]antiSMASH “regions” might include one or more BGCs; “Chr” states for “chromosome”. [2]antiSMASH annotation. [3]Beguining and end positions of the “region” as annotated by antiSMASH. [4]Know BGC with which the “region” contains highest proportion of similar genes, as annotated by antiSMASH. In bold type, compounds empirically identified as produced by *Embleya australiensis* MST-111070 (by Microbial Screening Technologies Pty. Ltd., data not shown); in normal type, BGCs with more than 50% similar genes; in italics, BGCs with under 50% similar genes or no known similar BGC. Underlined, BGCs highly conserved and essential for the full biological cycle of streptomycetes. [5] Assigned based upon similarity to the known polyoxypeptin BGC (BGC0001036). [6] Identified by blastn search and PKS similarity: 92% identity with Figure 5 from patent US 7,288,396 B2. [7]Assigned by antiSMASH as most similar to SAL-2242 (BGC0000546) and SapB (BGC0000551) from *Streptomyces coelicolor* A3(2). [8] Highly conserved BGC among streptomycetes.

Table S4: Highly conserved proteins between EEC1-like replicons.

BlastP hits of proteins encoded by *E. australiensis* MST-11070 against *Embleya* sp. NBC 00888 and *Embleya* sp. NBC 00896 with an identity on both replicons >95 %. Conserved domains and predicted functions informed by NCBI's CDD search, and by work conducted in this manuscript. Clusters of conserved genes are highlighted in grey.

Protein ID	%ID NBC00888	%ID NBC00896	Conserved Domains	Proposed Function
ctg2_731	95.4	96.3	LarA	Cell envelope biosynthesis
ctg2_737	96	96	PL-6	Carbohydrate transport and metabolism
ctg2_738	95.6	97.2	AfuC	Carbohydrate transport and metabolism
ctg2_739	97.6	97.2	UgpE	Carbohydrate transport and metabolism
ctg2_740	97.8	98.1	UgpA	Carbohydrate transport and metabolism
ctg2_827	97.2	97.2	DppB	Transport
ctg2_1763	99.3	97.9	Lrp	Regulation
ctg2_1888	100	99.3	ParA	Replication
ctg2_1891	100	98.1	Hypothetical	Replication
ctg2_2397	97.9	98.1	HyuB	Biosynthesis
ctg2_2402	97.1	95.4	FbaB	Carbohydrate transport and metabolism
ctg2_2511	96.6	96.6	DppB	Transport
ctg2_2548	96	95.3	IMPase	Phosphatase
ctg2_2987	95.4	95.5	AraH	Carbohydrate transport and metabolism
ctg2_2990	95.8	96.6	GlpR	Carbohydrate transport and metabolism
ctg2_3474	100	96.3	large tegument protein UL36	Unknown

Table S5: Features of *Embleya australiensis* MST-111070 EEC1 and large linear *Streptomyces* spp. replicons.

Summary of biochemical and genetic composition of EEC1-3 compared to the largest *Streptomyces* megaplasmid reported to date pSCL4 from *Streptomyces clavuligerus*[59]. Spearman's and Pearson's correlations are shown. Based on these classifications, neither EEC1 or pSCL4 fit the definition of a chromid.

	**EEC1**	**EEC2**	**EEC3**	**pSCL4**
**Species**	*E. australiensis*	*E. australiensis*	*E. australiensis*	*S. clavuligerus*
**Strain**	MST-111070	MST-111070	MST-111070	ATCC 27064
**Classification**	Secondary Chromosome	Mega plasmid	Plasmid	Unclassified, chromid-like
**Size**	4.2 Mb	0.3 Mb	0.2 Mb	1.8 Mb
**GC % (chromosomal)**	71 % (71 %)	69.1 (71 %)	69.5 (71%)	72 % (73%)
**Codon Bias (Correlation)**	Chromosomal (0.993/0.995)	Non-chromosomal (0.949/0.969)	Non-chromosomal (0.957/0.970)	Chromosomal (0.994/0.993)
**Functional Bias (Correlation)**	Chromosomal (0.972/0.984)	Non-chromosomal (0.543/0.456)	Non-chromosomal (0.499/0.325)	Chromosomal (0.938/0.917)
**BGC-density**	Higher than chromosome (~2x)	Higher than chromosome (~2x)	Higher than chromosome (~2x)	Higher than the chromosome (>2x)
**Replication System**	Unknown	Unknown	Plasmid	Plasmid
**Specificity**	Genus	Strain/species	Strain/species	Strain/species

Table S6: Analysis of other large replicons from *Streptomycetaceae*

Analysis of replicons encoded by other large replicons from *Streptomycetaceae*. The size of each replicon is shown in megabases, along with the % identity of each protein to those from the SCP1 replicon.

						SCP1 homology					
**Accession**	**Replicon**	**Genus**	**Species**	**Strain**	**Size (Mb)**	**ParA1**	**ParB1**	**ParA2**	**ParB2**	**Rep1**	**Rep2**
CP182423	EEC1	*Embleya*	*australiensis*	MST-11070	4.2	-	-	-	-	-	-
CP003229	pSCATT	*Streptantibioticus*	*cattleyicolor*	DSM 46488	1.8	-	-	-	-	-	-
CP045850	pSCL4	*Streptomyces*	*clavuligerus*	ATCC 27064	1.8	69	62.2	53.5	-	72.9	71.8
CP013220	"chromosome 2"	*Streptomyces*	*hygroscopicus*	KCTC 1717	1.9	87.1	60	55.8	44.4	59.3	60.9
CP109481	unnamed1	*Streptomyces*	sp.	NBC 01450	2.4	85.7	63.9	55.8	38.5	78.6	64.4

Table S7: P-values associated with codon usage correlation in *Embleya australiensis* MST-111070 (Figure 2c)

	EEC3	EEC2	EEC1	Chr.
**EEC3**	0	2.14E-58	1.80E-45	1.39E-39
**EEC2**	2.14E-58	0	1.86E-45	2.81E-39
**EEC1**	1.80E-45	1.86E-45	0	2.05E-63
**Chr.**	1.39E-39	2.81E-39	2.05E-63	0

Table S8: P-values associated with COG function correlation (Figure 2d)

	EEC1	EEC2	EEC3	Chr.
**EEC1**	0	2.62E-02	9.35E-02	6.06E-18
**EEC2**	2.62E-02	0	7.09E-06	2.51E-02
**EEC3**	9.35E-02	7.09E-06	0	1.21E-01
**Chr.**	6.06E-18	2.51E-02	1.21E-01	0

Table S9: P-values associated with codon usage correlation in *S. clavuligerus* ATCC 27064 (Figure S7)

	pSCL4	pSCL2	Chr.	pSCL1
**pSCL4**	0	1.39E-41	3.55E-63	2.67E-33
**pSCL2**	1.39E-41	0	4.01E-38	7.92E-35
**Chr.**	3.55E-63	4.01E-38	0	6.63E-32
**pSCL1**	2.67E-33	7.92E-35	6.63E-32	0

Table S10: P-values associated with COG function correlation (Figure S8)

	Chr.	pSCL1	pSCL2	pSCL4
**Chr.**	0	0.184685	0.000725	7.10E-11
**pSCL1**	0.184685	0	0.001802	0.109288
**pSCL2**	0.000725	0.001802	0	0.00012
**pSCL4**	7.10E-11	0.109288	0.00012	0

3. SUPPLEMENTARY FIGURES

Figure S1. Identification and analysis of terminal inverted repeats.

Blast analysis of the relationship between the terminal inverted repeats of the chromosomal contig (unitig_0) and the 4.2 Mb contig (unitig_1). A) Table of relevant blast HSPs; numbers in italics indicate that they are the last nucleotide of the contig, i.e. the HSP reaches the end of the contig. B) Graphical representation of the HSPs; each blast HSP is identified by a colour and number; the query and subject are represented by solid and dotted lines respectively.

A

Hit No.	query id	subject id	% identity	alignment length	mismatches	gap opens	q. start	q. end	s. start	s. End
1	unitig_0	unitig_0	99.99	21177	1	1	1	21176	*7117762*	7096586
2	unitig_0	unitig_1	99.94	33612	3	18	7084160	*7117762*	4184342	*4217944*
3	unitig_0	unitig_1	99.95	21184	1	9	1	21180	1	21179
4	unitig_0	unitig_1	99.95	21182	1	10	7096585	*7117762*	21176	1
5	unitig_0	unitig_1	99.95	21178	1	9	1	21176	*4217944*	4196774
6	unitig_1	unitig_1	99.91	21183	1	18	1	21176	*4217944*	4196773

B

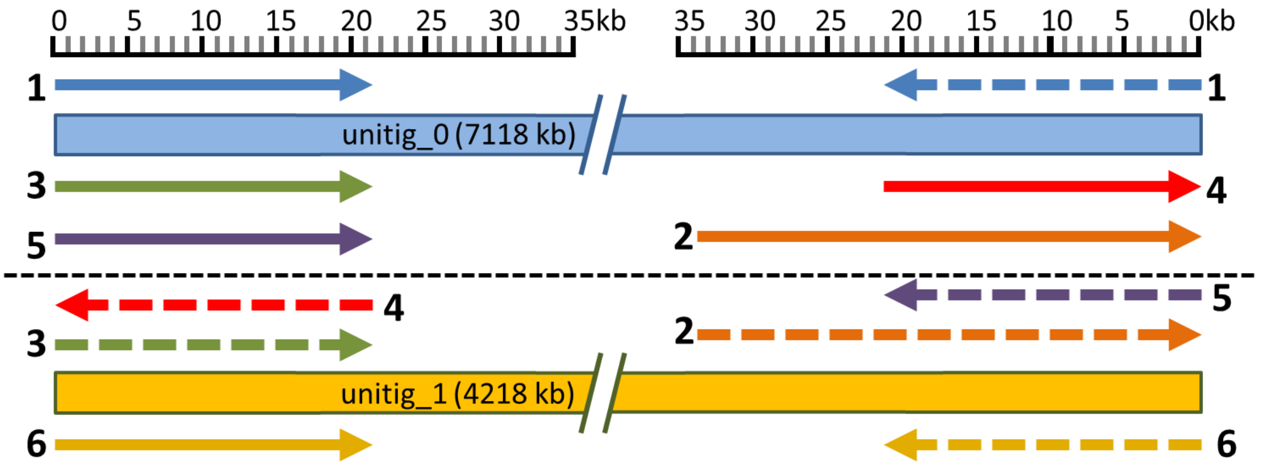

Figure S2. Alignment of Bionano molecules and DNA sequence assembly.

A. Overview of the alignment of BioNano molecules to the DNA sequence assembly, from left: chromosome, 4.2Mb contig (EEC1), the 302 kb linear EEC2, and circular plasmid EEC3. **B**. Full view of the alignment of BioNano molecules to the DNA sequence of EEC3, showing that because of the circular nature of this replicon, there are many Bionano molecules that extend outside the consensus DNA sequence used as reference, due to the different positions at which the plasmid molecule would have been opened during DNA extraction and BioNano assay.

A

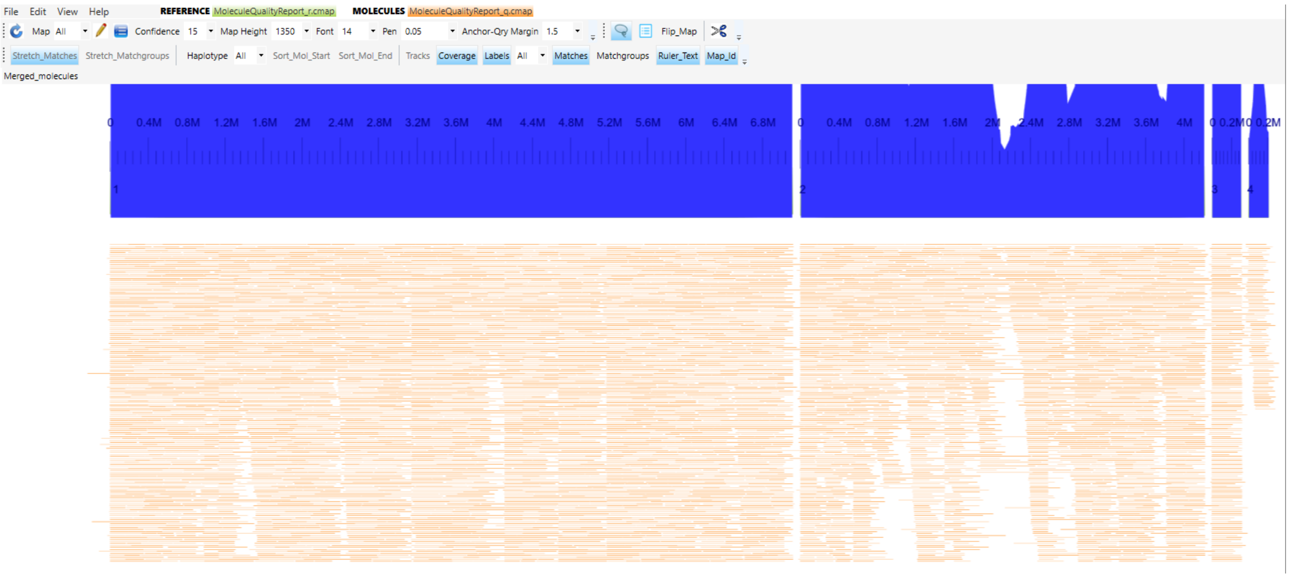

B

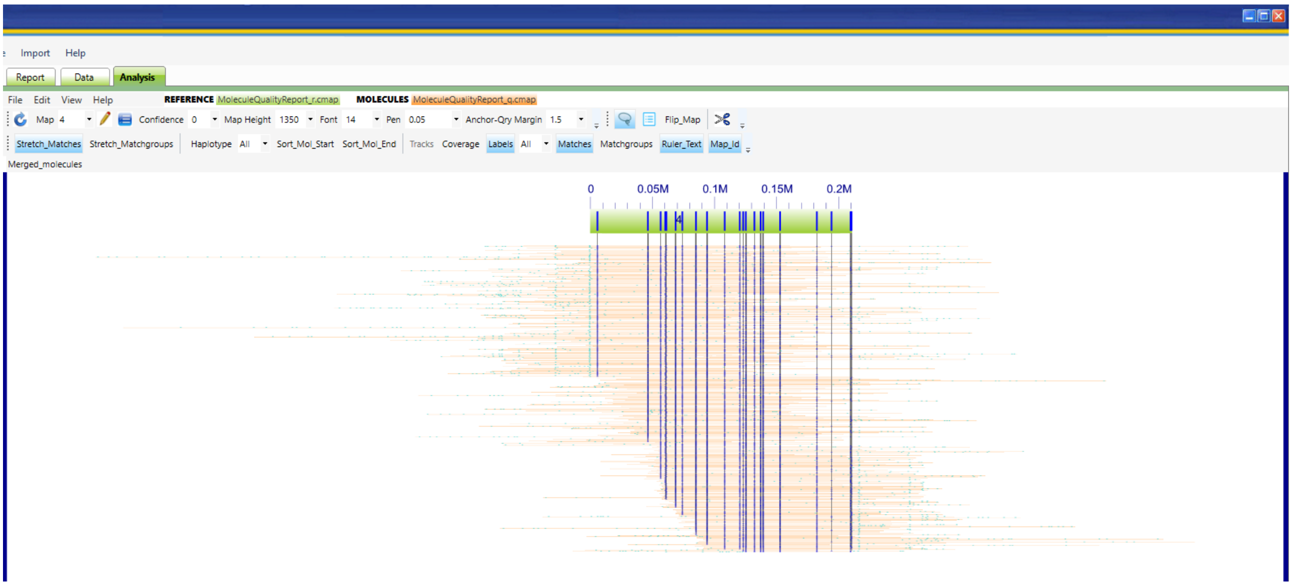

Figure S3. Alignment of Bionano and sequence genome assemblies.

Alignment of Bionano de-novo assembled contigs (blue) and DNA sequence assembly version 4 (green).

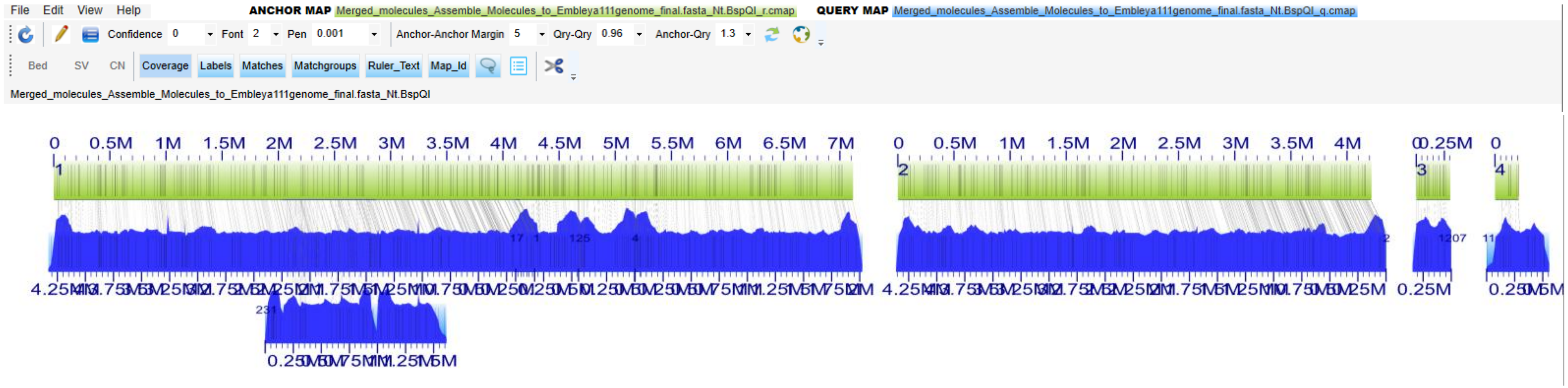

	Match_Id	Anchor_Id	AnchorStart	AnchorEnd	Size	Qry_Id	Qry_Start	Qry_End	Orient	Confdn
1	1	1	7,664	4,143,812	4,136,148	1	20	4,274,635	-	438.58
2	2	1	2,040,914	2,860,612	819,698	231	157,848	1,004,560	+	90.01
3	3	1	4,113,017	4,672,532	559,515	17	20	578,424	+	74.65
4	4	1	4,673,626	5,176,520	502,895	125	20	519,080	+	52.38
5	5	1	5,176,520	7,110,091	1,933,571	4	20	1,998,307	+	205.89
6	6	2	7,664	4,210,273	4,202,609	2	20	4,339,006	-	450.37
7	7	3	1,338	302,011	300,673	1207	20	310,441	-	36.4
8	8	4	5,647	210,490	204,843	11	80,320	291,203	+	21.86

Table of matches between Bionano de-novo assembled contigs and DNA-sequence assembly.

Figure S4. Overview of the alignment of the 211 Kb EEC3 circular plasmid with its corresponding BioNano contig.

The BioNano contig is more than double the size of the DNA-sequence contig; due to (and an indication of) the circular topology of this replicon, there are Bionano molecules that span in both directions of the purely artificial ends at which the circular sequence was opened (the stop codon of *parB*); it is also noticeable the repeated pattern of Bionano labels (vertical bars) in the Bionano contig (blue) at the right of the final match with the DNA-reference (green) as further support of the deducted circular topology.

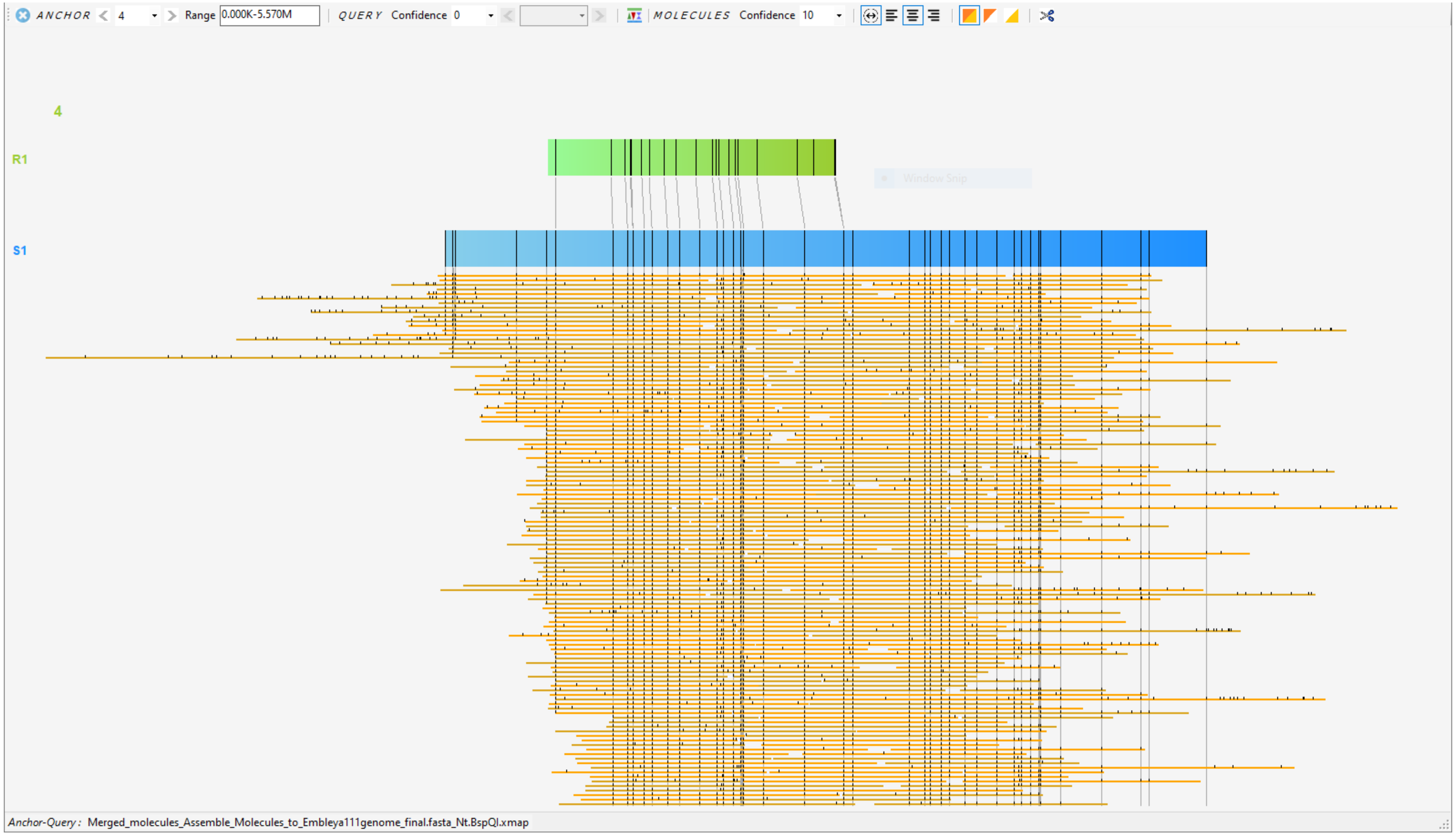

Figure S5. Phylogenetic analysis provided by TYGS for full genome and 16S analysis of *Embleya australiensis* MST-111070.

The final DNA assembly version 4 was uploaded to TYGS server [13] (on 2024-10-09 for the figures here shown). Phylogenetic tree based of full genome analysis (**A**) and 16S extended analysis offered by TYGS [13] with GGDC [15] (**B**) with only the genome of *E. australiensis* MST-111070 as query. This analysis initially revealed the membership of strain MST-111070 to the *Embleya* genus, and its close relationship with the genus *Yinghuangia*. (**A**) The values shown in blue are bootstrap values, in red are branch length. (**B**) The 16S tree was inferred under the GTR+CAT model and rooted by midpoint-rooting; the branches are scaled in terms of the expected number of substitutions per site; the numbers above the branches are support values when larger than 60% from Maximum likelihood (left) and maximum parsimony (right) bootstrapping. (modified from the automatic description provided by GGDC server). The *E. australiensis* MST-111070 genome is referred to as '111070v14genome_final' in the figure.

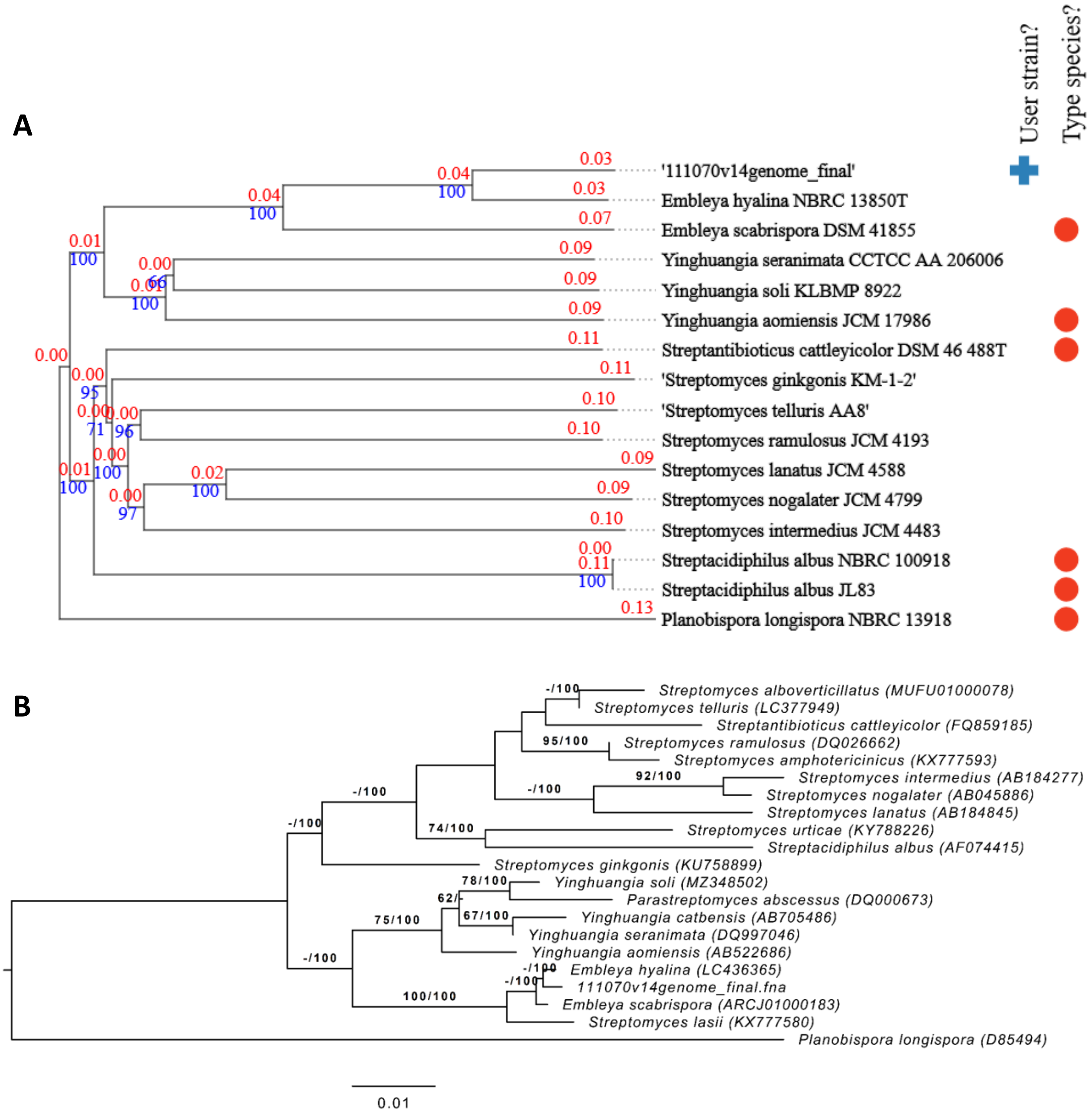

Figure S6. Extended phylogenetic analysis provided by TYGS for full genome and 16S analysis of *Embleya australiensis* MST-111070.

Next two pages.

The final DNA assembly version 4 (marked with red arrow) was uploaded to TYGS server [13] (on 2024-10-09 for the figures here shown) together with the genomes from NCBI databases identified as possibly belonging to *Embleya* and *Yinghuangia* genera (and other representative *Streptomyces* species). (**A**) Phylogenetic tree based of full genome analysis. Numbers above are bootstrap values. Blue cross at the right of the name indicates the genome as added as query by the user, while the red circle indicates that the strain is a type species of the genus. (**B**) 16S extended analysis offered by TYGS [13] with GGDC [15]. The tree was inferred under the GTR+CAT model and rooted by midpoint-rooting. The branches are scaled in terms of the expected number of substitutions per site. The numbers above the branches are support values when larger than 60% from Maximum likelihood (left) and maximum parsimony (right) bootstrapping. (modified from the automatic description provided by GGDC server).

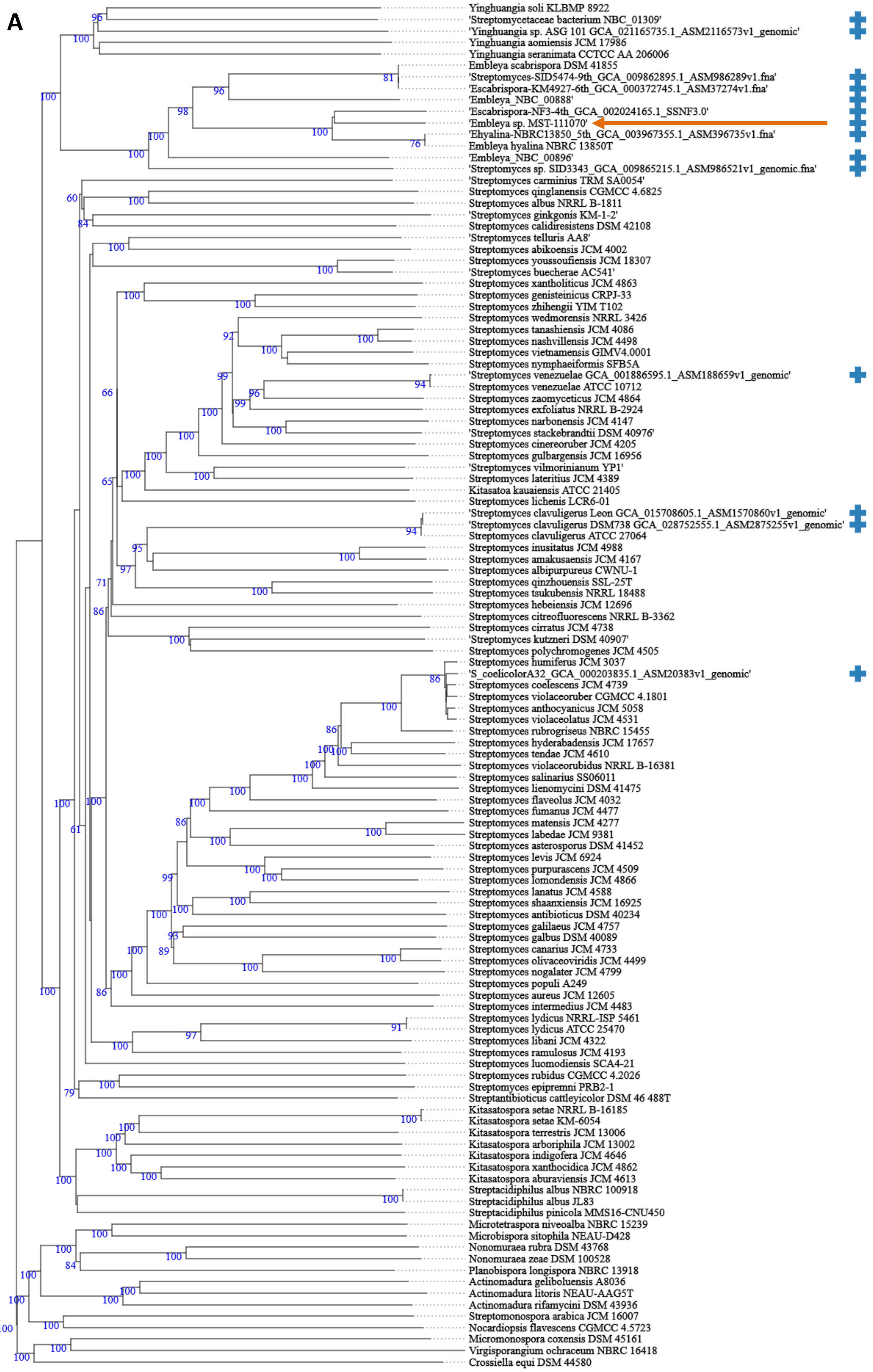
A
Yinghuangia soli KLBMP 8922
'Streptomycetaceae bacterium NBC_01309'
'Yinghuangia sp. ASG 101 GCA_021165735.1_ASM2116573v1_genomic'
Yinghuangia aomiensis JCM 17986
Yinghuangia seranimata CCTCC AA 206006
Embleya scabrispora DSM 41855
'Streptomyces-SID5474-9th_GCA_009862895.1_ASM986289v1.fna'
'Escabrispora-KM4927-6th_GCA_000372745.1_ASM37274v1.fna'
'Embleya_NBC_00888'
'Escabrispora-NF3-4th_GCA_002024165.1_SSNF3.0'
'Embleya sp. MST-111070'
'Ehyalina-NBRC13850_5th_GCA_003967355.1_ASM396735v1.fna'
Embleya hyalina NBRC 13850T
'Embleya_NBC_00896'
'Streptomyces sp. SID3343_GCA_009865215.1_ASM986521v1_genomic.fna'
'Streptomyces carminius TRM SA0054'
Streptomyces qinglanensis CGMCC 4.6825
Streptomyces albus NRRL B-1811
'Streptomyces ginkgonis KM-1-2'
Streptomyces calidiresistens DSM 42108
'Streptomyces telluris AA8'
Streptomyces abikoensis JCM 4002
Streptomyces youssoufiensis JCM 18307
'Streptomyces buecherae AC541'
Streptomyces xantholiticus JCM 4863
Streptomyces genisteinicus CRPJ-33
Streptomyces zhihengii YIM T102
Streptomyces wedmorensis NRRL 3426
Streptomyces tanashiensis JCM 4086
Streptomyces nashvillensis JCM 4498
Streptomyces vietnamensis GIMV4.0001
Streptomyces nymphaeiformis SFB5A
'Streptomyces venezuelae GCA_001886595.1_ASM188659v1_genomic'
Streptomyces venezuelae ATCC 10712
Streptomyces zaomyceticus JCM 4864
Streptomyces exfoliatus NRRL B-2924
Streptomyces narbonensis JCM 4147
'Streptomyces stackebrandtii DSM 40976'
Streptomyces cinereoruber JCM 4205
Streptomyces gulbargensis JCM 16956
'Streptomyces vilmorinianum YP1'
Streptomyces lateritius JCM 4389
Kitasatoa kauaiensis ATCC 21405
Streptomyces lichenis LCR6-01
'Streptomyces clavuligerus Leon GCA_015708605.1_ASM1570860v1_genomic'
'Streptomyces clavuligerus DSM738 GCA_028752555.1_ASM2875255v1_genomic'
Streptomyces clavuligerus ATCC 27064
Streptomyces inusitatus JCM 4988
Streptomyces amakusaensis JCM 4167
Streptomyces albipurpureus CWNU-1
Streptomyces qinzhouensis SSL-25T
Streptomyces tsukubensis NRRL 18488
Streptomyces hebeiensis JCM 12696
Streptomyces citreofluorescens NRRL B-3362
Streptomyces cirratus JCM 4738
'Streptomyces kutzneri DSM 40907'
Streptomyces polychromogenes JCM 4505
Streptomyces humiferus JCM 3037
'S_coelicolorA32_GCA_000203835.1_ASM20383v1_genomic'
Streptomyces coelescens JCM 4739
Streptomyces violaceoruber CGMCC 4.1801
Streptomyces anthocyanicus JCM 5058
Streptomyces violaceolatus JCM 4531
Streptomyces rubrogriseus NBRC 15455
Streptomyces hyderabadensis JCM 17657
Streptomyces tendae JCM 4610
Streptomyces violaceorubidus NRRL B-16381
Streptomyces salinarius SS06011
Streptomyces lienomycini DSM 41475
Streptomyces flaveolus JCM 4032
Streptomyces fumanus JCM 4477
Streptomyces matensis JCM 4277
Streptomyces labedae JCM 9381
Streptomyces asterosporus DSM 41452
Streptomyces levis JCM 6924
Streptomyces purpurascens JCM 4509
Streptomyces lomondensis JCM 4866
Streptomyces lanatus JCM 4588
Streptomyces shaanxiensis JCM 16925
Streptomyces antibioticus DSM 40234
Streptomyces galilaeus JCM 4757
Streptomyces galbus DSM 40089
Streptomyces canarius JCM 4733
Streptomyces olivaceoviridis JCM 4499
Streptomyces nogalater JCM 4799
Streptomyces populi A249
Streptomyces aureus JCM 12605
Streptomyces intermedius JCM 4483
Streptomyces lydicus NRRL-ISP 5461
Streptomyces lydicus ATCC 25470
Streptomyces libani JCM 4322
Streptomyces ramulosus JCM 4193
Streptomyces luomodiensis SCA4-21
Streptomyces rubidus CGMCC 4.2026
Streptomyces epipremni PRB2-1
Streptantibioticus cattleyicolor DSM 46 488T
Kitasatospora setae NRRL B-16185
Kitasatospora setae KM-6054
Kitasatospora terrestris JCM 13006
Kitasatospora arboriphila JCM 13002
Kitasatospora indigofera JCM 4646
Kitasatospora xanthocidica JCM 4862
Kitasatospora aburaviensis JCM 4613
Streptacidiphilus albus NBRC 100918
Streptacidiphilus albus JL83
Streptacidiphilus pinicola MMS16-CNU450
Microtetraspora niveoalba NBRC 15239
Microbispora sitophila NEAU-D428
Nonomuraea rubra DSM 43768
Nonomuraea zeae DSM 100528
Planobispora longispora NBRC 13918
Actinomadura geliboluensis A8036
Actinomadura litoris NEAU-AAG5T
Actinomadura rifamycini DSM 43936
Streptomonospora arabica JCM 16007
Nocardiopsis flavescens CGMCC 4.5723
Micromonospora coxensis DSM 45161
Virgisporangium ochraceum NBRC 16418
Crossiella equi DSM 44580

B

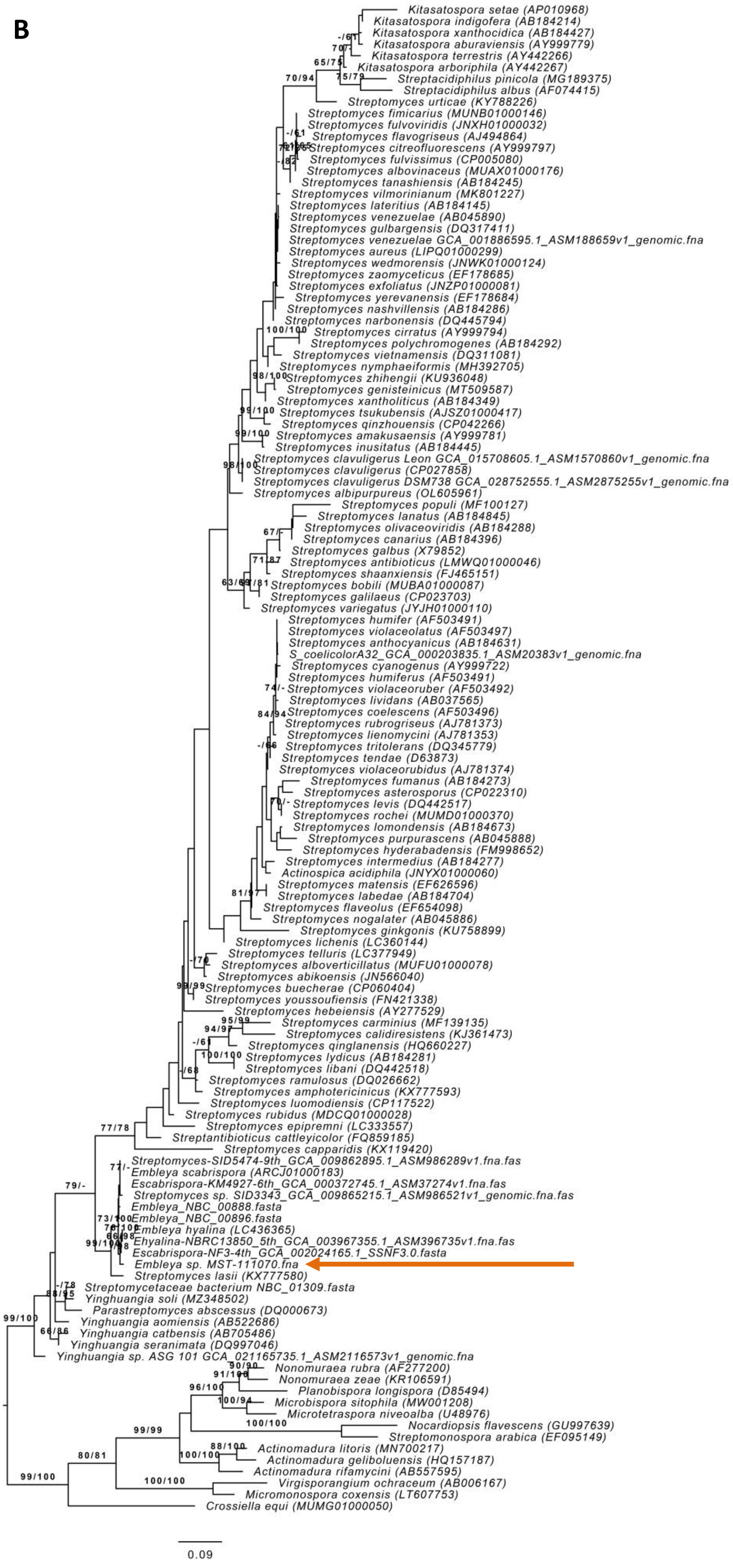
Kitasatospora setae (AP010968)
Kitasatospora indigofera (AB184214)
Kitasatospora xanthocidica (AB184427)
Kitasatospora aburaviensis (AY999779)
Kitasatospora terrestris (AY442266)
Kitasatospora arboriphila (AY442267)
Streptacidiphilus pinicola (MG189375)
Streptacidiphilus albus (AF074415)
Streptomyces urticae (KY788226)
Streptomyces fimicarius (MUNB01000146)
Streptomyces fulvoviridis (JNXH01000032)
Streptomyces flavogriseus (AJ494864)
Streptomyces citreofluorescens (AY999797)
Streptomyces fulvissimus (CP005080)
Streptomyces albovinaceus (MUAX01000176)
Streptomyces tanashiensis (AB184245)
Streptomyces vilmorinianum (MK801227)
Streptomyces lateritius (AB184145)
Streptomyces venezuelae (AB045890)
Streptomyces gulbargensis (DQ317411)
Streptomyces venezuelae GCA_001886595.1_ASM188659v1_genomic.fna
Streptomyces aureus (LIPQ01000299)
Streptomyces wedmorensis (JNWK01000124)
Streptomyces zaomyceticus (EF178685)
Streptomyces exfoliatus (JNZP01000081)
Streptomyces yerevanensis (EF178684)
Streptomyces nashvillensis (AB184286)
Streptomyces narbonensis (DQ445794)
Streptomyces cirratus (AY999794)
Streptomyces polychromogenes (AB184292)
Streptomyces vietnamensis (DQ311081)
Streptomyces nymphaeiformis (MH392705)
Streptomyces zhihengii (KU936048)
Streptomyces genisteinicus (MT509587)
Streptomyces xantholiticus (AB184349)
Streptomyces tsukubensis (AJSZ01000417)
Streptomyces qinzhouensis (CP042266)
Streptomyces amakusaensis (AY999781)
Streptomyces inusitatus (AB184445)
Streptomyces clavuligerus Leon GCA_015708605.1_ASM1570860v1_genomic.fna
Streptomyces clavuligerus (CP027858)
Streptomyces clavuligerus DSM738 GCA_028752555.1_ASM2875255v1_genomic.fna
Streptomyces albipurpureus (OL605961)
Streptomyces populi (MF100127)
Streptomyces lanatus (AB184845)
Streptomyces olivaceoviridis (AB184288)
Streptomyces canarius (AB184396)
Streptomyces galbus (X79852)
Streptomyces antibioticus (LMWQ01000046)
Streptomyces shaanxiensis (FJ465151)
Streptomyces bobili (MUBA01000087)
Streptomyces galilaeus (CP023703)
Streptomyces variegatus (JYJH01000110)
Streptomyces humifer (AF503491)
Streptomyces violaceolatus (AF503497)
Streptomyces anthocyanicus (AB184631)
S_coelicolorA32_GCA_000203835.1_ASM20383v1_genomic.fna
Streptomyces cyanogenus (AY999722)
Streptomyces humiferus (AF503491)
Streptomyces violaceoruber (AF503492)
Streptomyces lividans (AB037565)
Streptomyces coelescens (AF503496)
Streptomyces rubrogriseus (AJ781373)
Streptomyces lienomycini (AJ781353)
Streptomyces tritolerans (DQ345779)
Streptomyces tendae (D63873)
Streptomyces violaceorubidus (AJ781374)
Streptomyces fumanus (AB184273)
Streptomyces asterosporus (CP022310)
Streptomyces levis (DQ442517)
Streptomyces rochei (MUMD01000370)
Streptomyces lomondensis (AB184673)
Streptomyces purpurascens (AB045888)
Streptomyces hyderabadensis (FM998652)
Streptomyces intermedius (AB184277)
Actinospica acidiphila (JNYX01000060)
Streptomyces matensis (EF626596)
Streptomyces labedae (AB184704)
Streptomyces flaveolus (EF654098)
Streptomyces nogalater (AB045886)
Streptomyces ginkgonis (KU758899)
Streptomyces lichenis (LC360144)
Streptomyces telluris (LC377949)
Streptomyces alboverticillatus (MUFU01000078)
Streptomyces abikoensis (JN566040)
Streptomyces buecherae (CP060404)
Streptomyces youssoufiensis (FN421338)
Streptomyces hebeiensis (AY277529)
Streptomyces carminius (MF139135)
Streptomyces calidiresistens (KJ361473)
Streptomyces qinglanensis (HQ660227)
Streptomyces lydicus (AB184281)
Streptomyces libani (DQ442518)
Streptomyces ramulosus (DQ026662)
Streptomyces amphotericinicus (KX777593)
Streptomyces luomodiensis (CP117522)
Streptomyces rubidus (MDCQ01000028)
Streptomyces epipremni (LC333557)
Streptantibioticus cattleyicolor (FQ859185)
Streptomyces capparidis (KX119420)
Streptomyces-SID5474-9th_GCA_009862895.1_ASM986289v1.fna.fas
Embleya scabrispora (ARCJ01000183)
Escabrispora-KM4927-6th_GCA_000372745.1_ASM37274v1.fna.fas
Streptomyces sp. SID3343_GCA_009865215.1_ASM986521v1_genomic.fna.fas
Embleya_NBC_00888.fasta
Embleya_NBC_00896.fasta
Embleya hyalina (LC436365)
Ehyalina-NBRC13850_5th_GCA_003967355.1_ASM396735v1.fna.fas
Escabrispora-NF3-4th_GCA_002024165.1_SSNF3.0.fasta
Embleya sp. MST-111070.fna
Streptomyces lasii (KX777580)
Streptomycetaceae bacterium NBC_01309.fasta
Yinghuangia soli (MZ348502)
Parastreptomyces abscessus (DQ000673)
Yinghuangia aomiensis (AB522686)
Yinghuangia catbensis (AB705486)
Yinghuangia seranimata (DQ997046)
Yinghuangia sp. ASG 101 GCA_021165735.1_ASM2116573v1_genomic.fna
Nonomuraea rubra (AF277200)
Nonomuraea zeae (KR106591)
Planobispora longispora (D85494)
Microbispora sitophila (MW001208)
Microtetraspora niveoalba (U48976)
Nocardiopsis flavescens (GU997639)
Streptomonospora arabica (EF095149)
Actinomadura litoris (MN700217)
Actinomadura geliboluensis (HQ157187)
Actinomadura rifamycini (AB557595)
Virgisporangium ochraceum (AB006167)
Micromonospora coxensis (LT607753)
Crossiella equi (MUMG01000050)
0.09

Figure S7. Heatmap and hierarchical clustering of codon usage correlation for secondary replicons in *S. clavuligerus* ATCC 27064.

The figure was produced by Codoniser (this study). Correlation is calculated by Spearman's Rank.

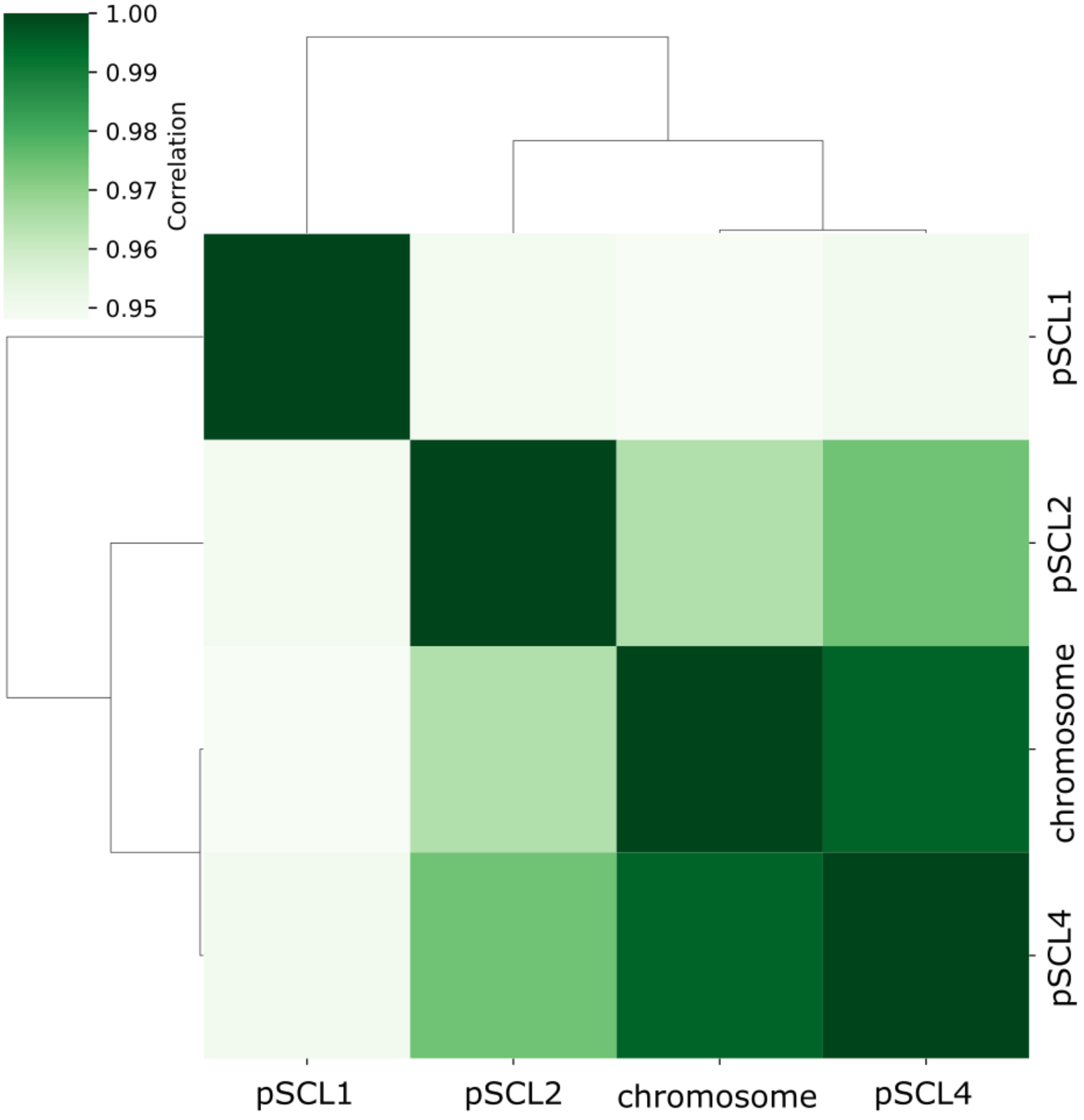

Figure S8. Heatmap and hierarchical clustering of COG utilisation correlation for secondary replicons in *S. clavuligerus* ATCC 27064.

The figure was produced by egger (this study). Correlation is calculated by Spearman's Rank.

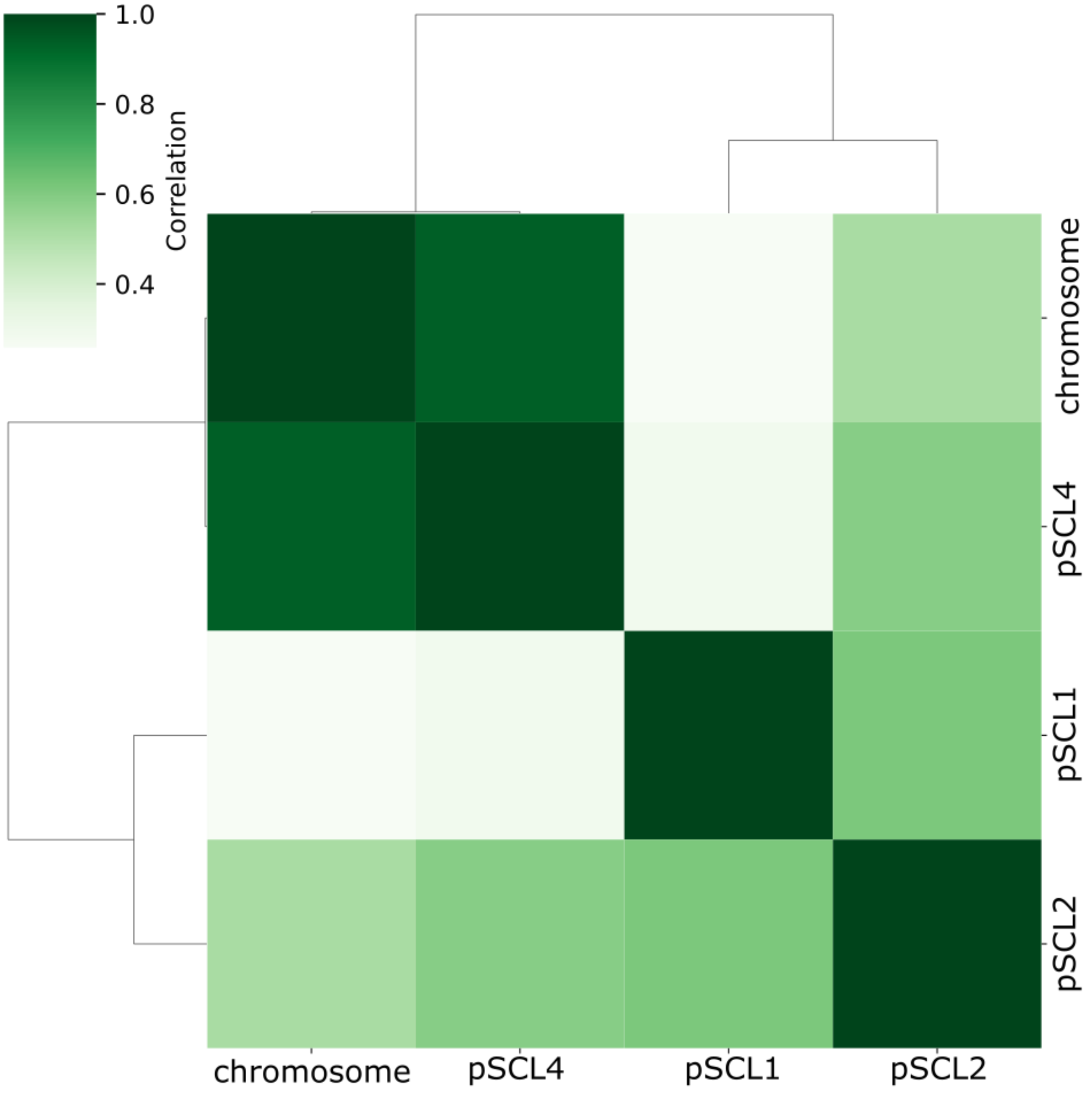

Figure S9. Original output of the annotation of putative specialised metabolism biosynthetic gene clusters by antiSMASH

Run on 2023-06-12 (continues on next two pages).

unitig_0_Illu_ext_both_ends

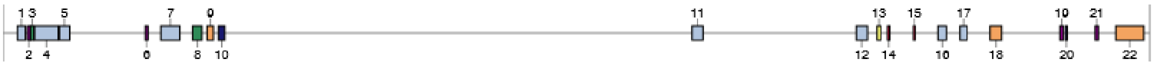

Region	Type	From	To	Most similar known cluster		Similarity
Region 1.1	T1PKS, linaridin	90,857	138,901	legonaridin	RiPP	41%
Region 1.2	terpene	150,651	171,443			
Region 1.3	CDPS	171,621	192,325			
Region 1.4	NRPS-like, NRPS, T1PKS	196,288	342,868	incednine	Polyketide	14%
Region 1.5	thiopeptide, LAP, T1PKS	349,551	414,110	paromomycin	Saccharide	7%
Region 1.6	terpene	882,668	901,353	dudomycin A	NRP	13%
Region 1.7	indole, NRPS, T1PKS	980,466	1,099,592	polyoxypeptin	NRP+Polyketide	75%
Region 1.8	NRP-metallophore, NRPS	1,177,888	1,235,978	griseobactin	NRP	92%
Region 1.9	T3PKS	1,266,421	1,307,485	alkylresorcinol	Polyketide	100%
Region 1.10	other	1,336,893	1,377,618	himastatin	NRP	12%
Region 1.11	NRPS, ladderane	4,277,131	4,344,826	ishigamide	NRP+Polyketide	100%
Region 1.12	NAPAA, T1PKS, hglE-KS	5,295,805	5,365,248	hexacosalactone A	Other	11%
Region 1.13	lanthipeptide-class-iv	5,425,093	5,448,404	labyrinthopeptin A2/labyrinthopeptin A1/labyrinthopeptin A3	RiPP:Lanthipeptide	40%
Region 1.14	NI-siderophore	5,489,809	5,503,424	schizokinen	Other	30%
Region 1.15	NI-siderophore	5,649,743	5,661,680			
Region 1.16	terpene, betalactone	5,803,706	5,856,397	hopene	Terpene	46%
Region 1.17	NRPS-like, betalactone	5,940,563	5,984,102	nybomycin	Other	55%
Region 1.18	T1PKS	6,127,601	6,199,142	neoabyssomicin/abyssomicin	Polyketide	31%
Region 1.19	terpene	6,559,664	6,581,427	polyoxypeptin	NRP+Polyketide	8%
Region 1.20	RiPP-like	6,595,549	6,606,349	persiamycin A	Polyketide:Type II polyketide	5%
Region 1.21	terpene	6,778,221	6,799,270			
Region 1.22	T1PKS	6,905,975	7,081,035	neoabyssomicin/abyssomicin	Polyketide	50%

unitig_1_Illu_Ext_both_ends

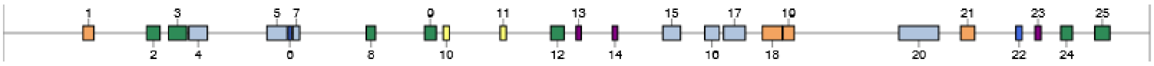

Region	Type	From	To	Most similar known cluster		Similarity
Region 2.1	PKS-like	294,513	335,535	rustmicin	Polyketide:Iterative type I polyketide	33%
Region 2.2	NRPS , NRPS-like	528,434	578,246	WS9326	NRP	10%
Region 2.3	NRPS , NRPS-like	608,894	675,912	himastatin	NRP	56%
Region 2.4	NRPS , betalactone	683,679	752,046	ohmyungsamycin A/ohmyungsamycin B	NRP	12%
Region 2.5	terpene , NRPS , aminocoumarin , ladderane , other , NRPS-like	971,229	1,044,800	acyldepsipeptide 1	NRP+Polyketide	26%
Region 2.6	RiPP-like	1,049,383	1,061,281			
Region 2.7	RRE-containing , butyrolactone	1,066,340	1,091,725			
Region 2.8	NAPAA	1,336,612	1,370,679	γ-poly-L-2,4-diaminobutyric acid	NRP	37%
Region 2.9	NRPS	1,550,635	1,593,139	triacsin C	Other	28%
Region 2.10	lanthipeptide-class-iv	1,619,382	1,641,973	ulleungmycin	NRP	5%
Region 2.11	lanthipeptide-class-iii	1,828,683	1,851,442	SAL-2242	RiPP:Lanthipeptide	55%
Region 2.12	NRPS	2,015,110	2,063,485	aurantimycin A	NRP+Polyketide	5%
Region 2.13	terpene	2,107,635	2,127,304			
Region 2.14	terpene	2,241,370	2,260,810			
Region 2.15	NRPS , T1PKS , NRPS-like	2,426,374	2,492,278	ECO-0501	Polyketide	19%
Region 2.16	NRPS , lanthipeptide-class-ii	2,581,031	2,634,701			
Region 2.17	NI-siderophore , NRPS , NRPS-like	2,650,507	2,730,644	peucechelin	NRP	25%
Region 2.18	T2PKS	2,792,990	2,865,499	spore pigment	Polyketide	83%
Region 2.19	T3PKS	2,869,003	2,910,154	R1128	Polyketide	21%
Region 2.20	T1PKS , ladderane , arylpolyene , NRPS-like	3,295,026	3,442,212	akaeolide	Polyketide	16%
Region 2.21	hglE-KS	3,522,775	3,573,744			
Region 2.22	lassopeptide	3,725,618	3,748,424			
Region 2.23	terpene	3,796,308	3,817,516	ebelactone	Polyketide	5%
Region 2.24	NRPS-like	3,890,646	3,933,120			
Region 2.25	NRPS-like	4,015,181	4,071,452	ecumicin	NRP	10%

unitig_2

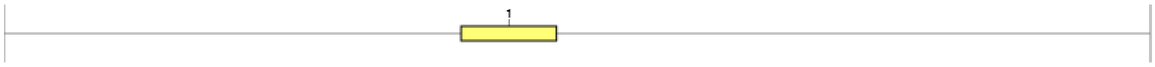

Region	Type	From	To	Most similar known cluster		Similarity
Region 3.1	lanthipeptide-class-i	120,682	145,886			

unitig3_circular

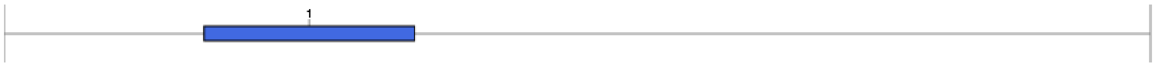

Region	Type	From	To	Most similar known cluster		Similarity
Region 4.1	ranthipeptide	36,794	75,454	desulfoclethramycin/clethramycin	Other	4%

Figure S10: Cumulative GC-skew of *Embleya australiensis* MST-111070 replicons.

Showing the cumulative GC-skew for a) the chromosome, b) EEC1, c) EEC2 and, d) EEC3. The location of *parA* homologues is indicated along with their position in the genome. Step and window sizes were calculated as a percentage of the replicon size.

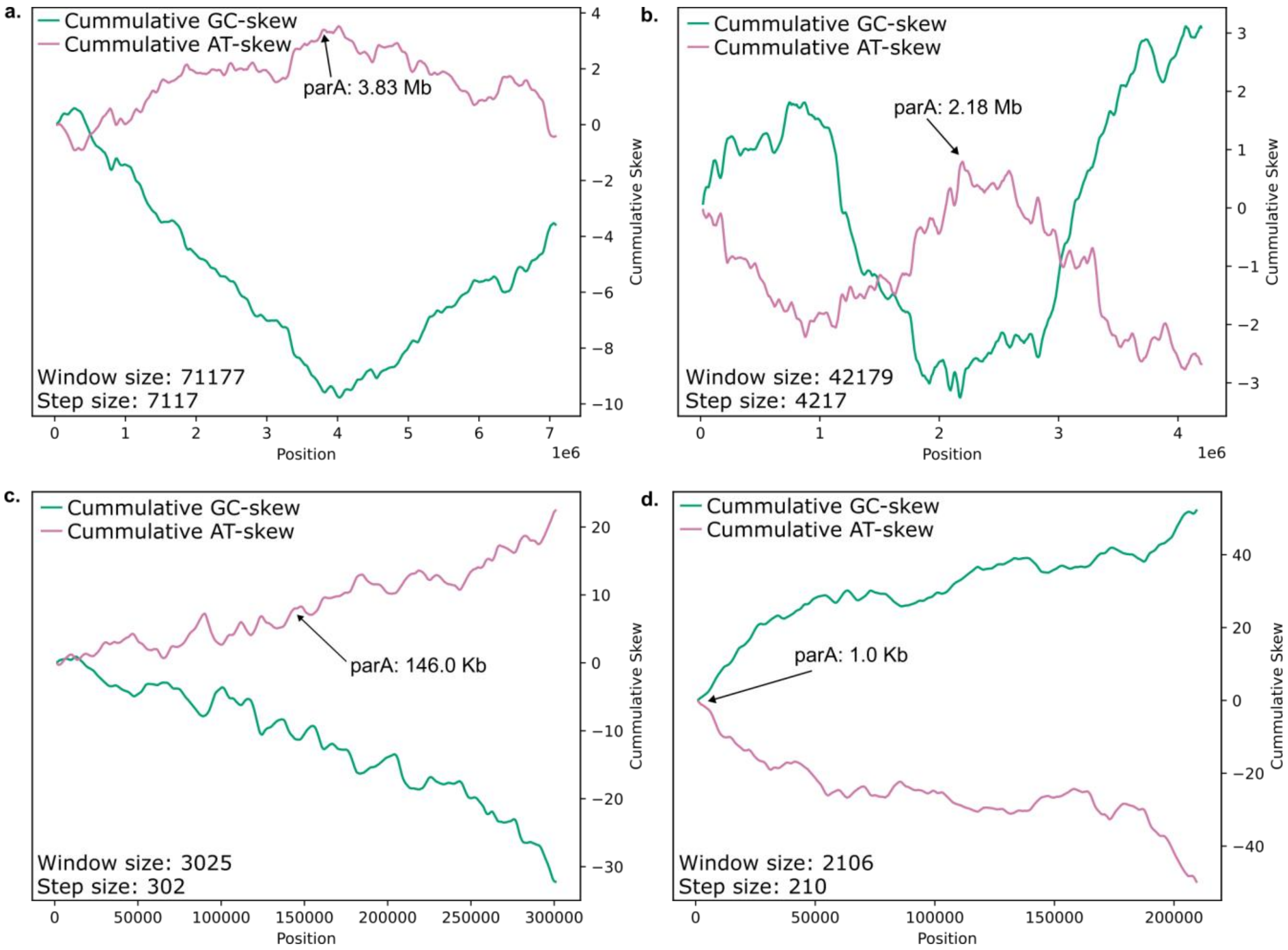

Figure S11. antiSMASH annotation of the large secondary replicon of high-quality genome assemblies of other *Embleya* species.

View of the antiSMASH annotation of the large secondary replicon of *Embleya* sp. NBC 00888 (5.7 Mb, top) and *Embleya* sp. NBC 00896 (3.1 Mb, bottom), showing the presence of the highly conserved and essential BGCs for the spore pigment and SapB (not readily identified by antiSMASH in NCB 00896 in any of its replicons) in this secondary replicon, instead of in the chromosome as it is the case in all other genera of the *Streptomycetaceae* family. For the analysis, we uploaded to antiSMASH the annotated NCBI RefSeq assembly files GCF_045207655.1 and GCF_045207785.1.

NZ_CP108785.1 (Embleya sp. NBC_00888)

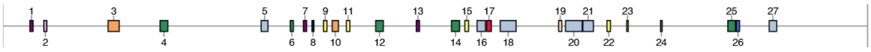

Region	Type	From	To	Most similar known cluster		Similarity
Region 2.1	terpene	179,678	200,661	mycotrienin I	NRP+Polyketide	7%
Region 2.2	phenazine	271,236	291,739	pyocyanine	Other	71%
Region 2.3	T1PKS	697,343	771,720	spectinabilin/orinocin/SNF4435C/ SNF4435D	Polyketide:Modular type I polyketide	90%
Region 2.4	NRPS-like , NRPS	1,042,149	1,093,548			
Region 2.5	arylpolyene , NRPS-like	1,712,211	1,757,005	calicheamicin	Polyketide	8%
Region 2.6	CDPS	1,904,899	1,925,612			
Region 2.7	terpene	1,992,235	2,013,305	ishigamide	NRP+Polyketide	11%
Region 2.8	hydrogen-cyanide	2,049,038	2,062,163			
Region 2.9	lanthipeptide-class-iv	2,125,344	2,147,956	ulleungmycin	NRP	5%
Region 2.10	T3PKS	2,183,139	2,224,251	napyradiomycin	Terpene	7%
Region 2.11	lanthipeptide-class-iii	2,277,354	2,299,966	SapB	RiPP:Lanthipeptide	75%
Region 2.12	NRPS	2,471,282	2,521,702			
Region 2.13	terpene	2,740,358	2,761,458			
Region 2.14	NRPS	2,973,699	3,027,354	stenothricin	NRP:Cyclic depsipeptide	9%
Region 2.15	lanthipeptide-class-i	3,062,251	3,086,787			
Region 2.16	NRPS , lanthipeptide-class-ii	3,143,339	3,198,749	leinamycin	NRP+Polyketide:Modular type I polyketide+Polyketide:Trans-AT type I polyketide	4%
Region 2.17	NI-siderophore	3,206,430	3,238,052	peucechelin	NRP	20%
Region 2.18	NRPS-like , T2PKS	3,296,406	3,401,918	spore pigment	Polyketide	83%
Region 2.19	indole	3,683,838	3,704,992			
Region 2.20	NRP-metallophore , NRPS , terpene , NAPAA	3,729,075	3,839,439	madurastatin A2/madurastatin E1/ madurastatin F/madurastatin G1/ madurastatin A1	NRP	47%
Region 2.21	hglE-KS , NRPS	3,844,397	3,914,475	triacsin C	Other	28%
Region 2.22	lanthipeptide-class-iv	4,004,293	4,026,980			
Region 2.23	ectoine	4,135,088	4,145,486	ectoine	Other	100%
Region 2.24	butyrolactone	4,359,055	4,369,978			
Region 2.25	NRPS	4,804,906	4,855,300	bonnevillamide D/bonnevillamide E	NRP	10%
Region 2.26	linaridin	4,861,744	4,882,661	legonaridin	RiPP	41%
Region 2.27	terpene , NRPS , T1PKS	5,079,299	5,128,694	dutomycin	Polyketide	4%

NZ_CP108777.1 (Embleya sp. NBC_00896)

Region	Type	From	To	Most similar known cluster		Similarity
Region 2.1	T3PKS , NRPS	433,330	499,867	feglymycin	NRP	36%
Region 2.2	NI-siderophore	890,414	922,039	peucechelin	NRP	20%
Region 2.3	RiPP-like	942,644	953,840			
Region 2.4	lanthipeptide-class-i	993,965	1,018,408			
Region 2.5	terpene	1,056,104	1,077,156			
Region 2.6	terpene , NRPS	1,078,830	1,151,878	neocarazostatin A	Other	60%
Region 2.7	T3PKS , NAPAA	1,195,911	1,247,420	napyradiomycin	Terpene	7%
Region 2.8	ectoine	1,298,872	1,309,270	ectoine	Other	100%
Region 2.9	terpene	1,311,164	1,332,270			
Region 2.10	hydrogen-cyanide	1,794,792	1,807,731	aborycin	RiPP	21%
Region 2.11	lanthipeptide-class-ii	1,858,317	1,881,226			
Region 2.12	T2PKS	1,929,028	2,001,585	spore pigment	Polyketide	83%
Region 2.13	NRPS , NRP-metallophore	2,185,839	2,269,601	madurastatin D1/madurastatin D2/(-)-Madurastatin C1	NRP	8%
Region 2.14	hydrogen-cyanide	2,462,196	2,475,223			

Figure S12. Artemis Comparison Tool view of the genome sequence comparison.

Artemis Comparison Tool view of the genome sequence comparison between (**A**) *Embleya australiensis* MST-111070 and *Embleya scabrispora* NF3 (minimum Score cut-off of 3795 was set to facilitate visualisation of most relevant similarities) and (**B**) strain 111070 and *Embleya hyalina* (minimum Score cut-off of 3344).

A **B**

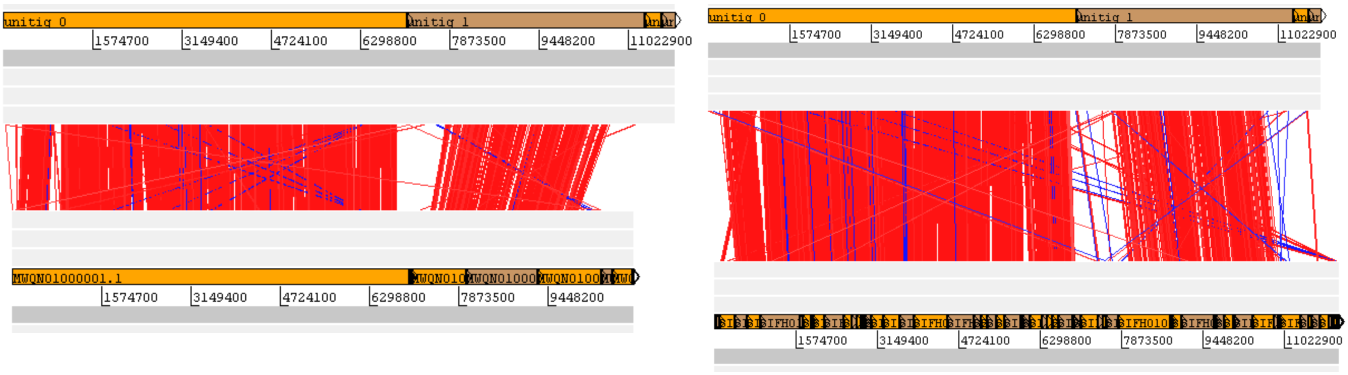

Figure S13. Alignment of the genome assemblies of *Embleya australiensis* MST-111070 with available genome assemblies from other *Embleya* strains.

Alignment with Mauve of the genome assemblies of *E. australiensis* MST-111070 with all the available genome assemblies for *Embleya* strains. The "backbone colour" representation has been selected, meaning that similar genome regions shared across all genomes are highlighted with mauve colour, while regions shared between two or three are marked with other colours. Most of the *E. australiensis* MST-111070 genome is shared across all three other assemblies, that includes most of the 4.2 Mb contig, indicating that this contig is not just a strain or species-specific extrachromosomal replicon but a genus shared characteristic.

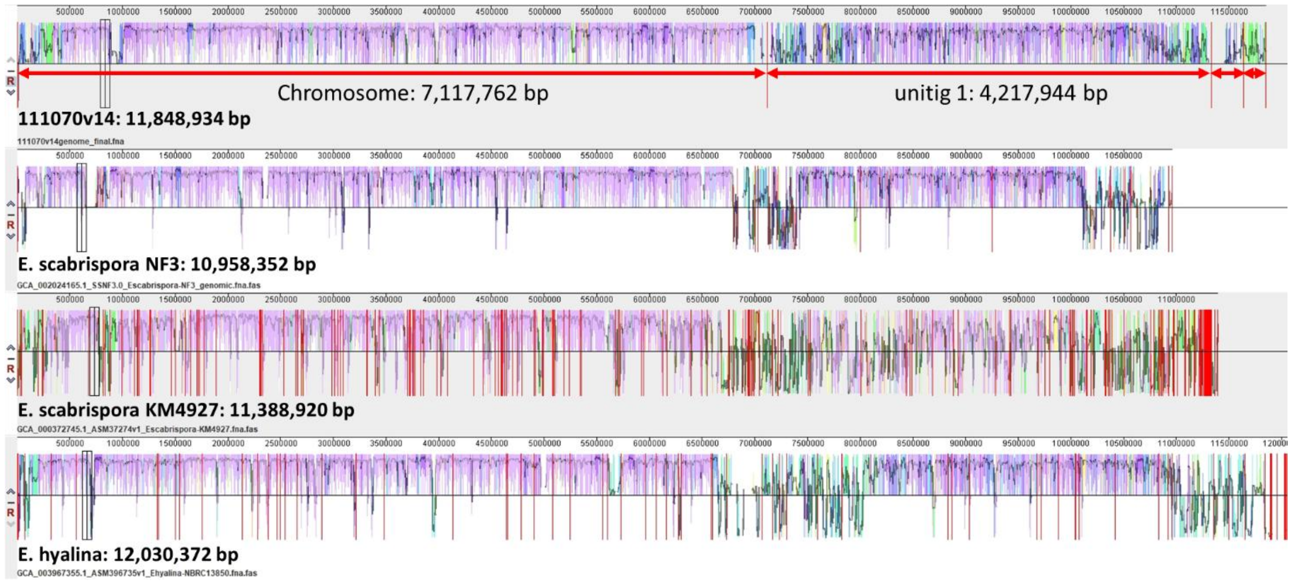
Chromosome: 7,117,762 bp
unitig 1: 4,217,944 bp
111070v14: 11,848,934 bp
111070v14genome_final.fna
E. scabrispora NF3: 10,958,352 bp
GCA_002024165.1_SSNF3.0_Escabrispora-NF3_genomic.fna.fas
E. scabrispora KM4927: 11,388,920 bp
GCA_000372745.1_ASM37274v1_Escabrispora-KM4927.fna.fas
E. hyalina: 12,030,372 bp
GCA_003967355.1_ASM396735v1_Ehyalina-NBRC13850.fna.fas

4. REFERENCES

1. **Rutherford K, Parkhill J, Crook J, Horsnell T, Rice P, Rajandream M-A, Barrell B**. Artemis: sequence visualization and annotation. *Bioinformatics* 2000;16:944–945, doi:10.1093/bioinformatics/16.10.944.

2. **Carver TJ, Rutherford KM, Berriman M, Rajandream M-A, Barrell BG, Parkhill J**. ACT: the Artemis comparison tool. *Bioinformatics* 2005;21:3422–3423, doi:10.1093/bioinformatics/bti553.

3. **Staden R**. The staden sequence analysis package. *Mol Biotechnol* 1996;5:233, doi:10.1007/BF02900361.

4. **Staden R, Beal KF, Bonfield JK**. The Staden Package, 1998. In: Misener S, Krawetz SA (editors). *Bioinformatics Methods and Protocols*. Totowa, NJ: Humana Press. pp. 115–130 ISBN 978-1-59259-192-3.

5. **Altschul SF, Madden TL, Schäffer AA, Zhang J, Zhang Z, Miller W, Lipman DJ**. Gapped BLAST and PSI-BLAST: a new generation of protein database search programs. *Nucleic Acids Res* 1997;25:3389–3402, doi:10.1093/nar/25.17.3389.

6. **Santiago-Sotelo P, Ramirez-Prado JH**. prfectBLAST: a platform-independent portable front end for the command terminal BLAST+ stand-alone suite. *BioTechniques* 2012;53:299–300, doi:10.2144/000113953.

7. **Blin K, Shaw S, Augustijn HE, Reitz ZL, Biermann F, Alanjary M, Fetter A, Terlouw BR, Metcalf WW, Helfrich EJN, van Wezel GP, Medema MH, Weber T**. antiSMASH 7.0: new and improved predictions for detection, regulation, chemical structures and visualisation. *Nucleic Acids Research* 2023;gkad344, doi:10.1093/nar/gkad344.

8. **Aziz RK, Bartels D, Best AA, DeJongh M, Disz T, Edwards RA, Formsma K, Gerdes S, Glass EM, Kubal M, Meyer F, Olsen GJ, Olson R, Osterman AL, Overbeek RA, McNeil LK, Paarmann D, Paczian T, Parrello B, Pusch GD, Reich C, Stevens R, Vassieva O, Vonstein V, Wilke A, Zagnitko O**. The RAST Server: Rapid Annotations using Subsystems Technology. *BMC Genomics* 2008;9:75, doi:10.1186/1471-2164-9-75.

9. **Overbeek R, Olson R, Pusch GD, Olsen GJ, Davis JJ, Disz T, Edwards RA, Gerdes S, Parrello B, Shukla M, Vonstein V, Wattam AR, Xia F, Stevens R**. The SEED and the Rapid Annotation of microbial genomes using Subsystems Technology (RAST). *Nucl Acids Res* 2014;42:D206–D214, doi:10.1093/nar/gkt1226.

10. **Hyatt D, Chen G-L, Locascio PF, Land ML, Larimer FW, Hauser LJ**. Prodigal: prokaryotic gene recognition and translation initiation site identification. *BMC Bioinformatics* 2010;11:119, doi:10.1186/1471-2105-11-119.

11. **Darling AE, Mau B, Perna NT**. progressiveMauve: Multiple Genome Alignment with Gene Gain, Loss and Rearrangement. *PLOS ONE* 2010;5:e11147, doi:10.1371/journal.pone.0011147.

12. **Simão FA, Waterhouse RM, Ioannidis P, Kriventseva EV, Zdobnov EM**. BUSCO: assessing genome assembly and annotation completeness with single-copy orthologs. *Bioinformatics* 2015;31:3210–3212, doi:10.1093/bioinformatics/btv351.

13. **Meier-Kolthoff JP, Göker M**. TYGS is an automated high-throughput platform for state-of-the-art genome-based taxonomy. *Nature Communications* 2019;10:2182, doi:10.1038/s41467-019-10210-3.

14. **Meier-Kolthoff JP, Auch AF, Klenk H-P, Göker M**. Genome sequence-based species delimitation with confidence intervals and improved distance functions. *BMC Bioinformatics* 2013;14:60, doi:10.1186/1471-2105-14-60.

15. **Meier-Kolthoff JP, Carbasse JS, Peinado-Olarte RL, Göker M**. TYGS and LPSN: a database tandem for fast and reliable genome-based classification and nomenclature of prokaryotes. *Nucleic Acids Research* 2022;50:D801–D807, doi:10.1093/nar/gkab902.

16. **Buchfink B, Xie C, Huson DH**. Fast and sensitive protein alignment using DIAMOND. *Nat Methods* 2015;12:59–60, doi:10.1038/nmeth.3176.
